# Metabolomic Strategies to Improve Chemical Information from OSMAC Studies of Endophytic Fungi

**DOI:** 10.3390/metabo13020236

**Published:** 2023-02-05

**Authors:** Fernanda Motta Ribeiro da Silva, Gecele Matos Paggi, Flávia Roberta Brust, Alexandre José Macedo, Denise Brentan Silva

**Affiliations:** 1 Laboratory of Natural Products and Mass Spectrometry (LaPNEM), Federal University of Mato Grosso do Sul, Campo Grande 79070-900, Brazil; 2 Laboratory of Ecology and Evolutionary Biology (LEBio), Institute of Biosciences, Federal University of Mato Grosso do Sul, Campo Grande 79070-900, Brazil; 3 Biofilms and Diversity Laboratory, Faculty of Pharmacy and Biotechnology Center, Federal University of Rio Grande do Sul, Porto Alegre 91501-970, Brazil

**Keywords:** metabolomics, OSMAC, molecular networking, bioprospection

## Abstract

Metabolomics strategies are important tools to get holistic chemical information from a system, but they are scarcely applied to endophytic fungi to understand their chemical profiles of biosynthesized metabolites. Here *Penicillium* sp. was cultured using One Strain Many Compounds (OSMAC) conditions as a model system to demonstrate how this strategy can help in understanding metabolic profiles and determining bioactive metabolites with the application of metabolomics and statistical analyses, as well as molecular networking. *Penicillium* sp. was fermented in different culture media and the crude extracts from mycelial biomass (CEm) and broth (CEb) were obtained, evaluated against bacterial strains (*Staphylococcus aureus* and *Pseudomonas aeruginosa*), and the metabolomic profiles by LC-DAD-MS were obtained and chemometrics statistical analyses were applied. The CEm and CEb extracts presented different chemical profiles and antibacterial activities; the highest activities observed were against *S. aureus* from CEm (MIC = 16, 64, and 128 µg/mL). The antibacterial properties from the extracts were impacted for culture media from which the strain was fermented. From the Volcano plot analysis, it was possible to determine statistically the most relevant features for the antibacterial activity, which were also confirmed from biplots of PCA as strong features for the bioactive extracts. These compounds included **75** (13-oxoverruculogen isomer), **78** (austalide P acid), **87** (austalide L or W), **88** (helvamide), **92** (viridicatumtoxin A), **96** (austalide P), **101** (dihydroaustalide K), **106** (austalide k), **110** (spirohexaline), and **112** (pre-viridicatumtoxin). Thus, these features included diketopiperazines, meroterpenoids, and polyketides, such as indole alkaloids, austalides, and viridicatumtoxin A, a rare tetracycline.

## 1. Introduction

The search for natural products with biological activities from endophytic fungi is imminent, due to their remarkable ability to biosynthesize compounds with high levels of structural complexity and prominent pharmacological properties [1,2]. Endophytic fungi have a valuable biosynthetic arsenal for the biotechnological production of promising compounds for the pharmaceutical, agricultural, and food industries; moreover, they are compatible with Sustainable Development Goals [3]. In addition, microorganisms are important sources of molecules that can act against human and animal diseases, such as cancer and bacterial infections. Thus, they are notable for the research and development of new antibacterials, which are drugs in high demand due to the current global crisis of bacterial infections [4].

The regulation of the biosynthesis of natural products is complex and the conditions under which the microorganisms are cultivated can drastically influence the production of metabolites [5]. The application of new strategies to get bioactive compounds has been an alternative to improve the current natural product research and access new products. Traditional bioprospection studies from fungi require large-scale growth to produce extracts and often can lead to the re-isolation of known substances [6]. Microorganisms have an untapped biosynthetic arsenal due to the typical growth conditions in the laboratory, and several methodologies to increase the chemical diversity by the induction of silenced gene clusters have been described, such as co-cultivation, the addition of elicitor molecules, and One Strain Many Compounds (OSMAC) strategy [7]. The OSMAC concept is based on the hypothesis that changes in cultivation parameters can modify the biosynthetic profiles of microorganism strains that can increase the variability of compound production by a single strain and lead to the discovery of new compounds or increase the production of a specific metabolite [8]. OSMAC strategy is gaining prominence due to the low investment required and its promising results.

Metabolomics can be defined as a comprehensive analysis of metabolites to get the metabolic information of low weight (<1000 Da) from an organism or biological system [9]. The metabolomics associated with chemometric tools is a useful tool to get holist chemical information [10] from microorganisms, resulting in a broad view of metabolite production, as well as assisting the appointments of metabolic pathways, global metabolism of cells, and giving a direction for the selection of metabolites of interest, such as bioactive metabolites for isolation procedures [11]. In addition, metabolomics can contribute to different research fields, for example, fungal classification and identification, metabolic pathways, the comprehension of plant-fungal chemical interactions, and the discovery of fungal natural products [12]. However, fungal metabolomics is still under development, and the databases of fungal metabolites remain singular and incomplete [9].

Given that there is still no consensus for an initial and wide investigation of metabolite production capacity by endophytic fungi and that the choice of culture medium influences this biosynthetic production from the microorganisms, here we aimed to evaluate the metabolites produced by the strain *Penicillium* sp. 5MP2F4 applying the OSMAC strategy and screening the antibacterial and antibiofilm activities. Additionally, we aimed to get broad chemical information by metabolomics and chemometrics tools.

## 2. Materials and Methods

### 2.1. Fungal Material and Identification of Strain

The strain *Penicillium* sp. 5MP2F4 (Figure S1, Supporting Information) was isolated in May 2019 from leaves of *Bromelia balansae* Mez (Bromeliaceae), a bromeliad species occurring in the ironstone outcrops of the Pantanal biome in Brazil. The fungal strain was identified according to morphological traits and ITS rDNA sequence analysis, as reported by Cassemiro et al. [13]. The sequence data were submitted to GenBank with accession n° OP879817. The strain was deposited at the library of the Federal University of Mato Grosso do Sul. The study was registered under the number AE8625B in the National Genetic Heritage Management System (SisGen).

### 2.2. OSMAC-Based Cultivation of Fungi

The strain was incubated in five different culture media to activate biosynthetic gene clusters [8]. The submerged culture media were the following: PDB (Potato Dextrose Broth—potato infusion (infusion from 200 g/L of potatoes) and 20 g/L of dextrose); MEB (Malt Extract Broth—dextrose 6 g/L, malt extract 6 g/L, maltose 1.8 g/L, and yeast extract 1.2 g/L); YPD (Yeast extract Peptone Dextrose—yeast extract 10 g/L, peptone 20 g/L, and dextrose 20 g/L); SAB (Sabouraud—peptone 10 g/L, and dextrose 40 g/L); CZA (Czapek—sucrose 30 g/L, NaNO_3_ 3 g/L, K_2_HPO_4_ 1 g/L, KCl 0.5 g/L, MgSO_4_ 0.5 g/L, and FeSO_4_ 0.01 g/L).

The fungus 5MP2F4 was initially cultivated on PDA (Potato Dextrose Agar—Infusion broth of 200 g/L potatoes, 20 g/L dextrose, and 15 g/L agar) and then a pre-inoculum was prepared on submerged PDB medium and cultured under static conditions for 7 days at 28 ± 2 °C. The OSMAC experiment was performed in submerged culture media, under static conditions, in the absence of light, and in Erlenmeyer flasks, each containing 150 mL of medium and 5 mL of pre-inoculum for inoculation. The experiments were performed in triplicate incubated for 16 days, and subsequently, they were pooled to produce one sample for each culture medium.

### 2.3. Extraction from Broth and Mycelial Biomass

From liquid cultures of OSMAC experiments, the crude extracts (CE) from broth (CEb) and mycelial biomass (CEm) were obtained after fermentation for 16 days. The mycelial biomass was separated from the broth by filtration. The broth was extracted by liquid-liquid extraction using ethyl acetate containing 1% formic acid, and the compounds with higher affinity for the organic phase were extracted based on their partition coefficient. The organic phase was separated and concentrated by rotary evaporation (Buchi, São Paulo, Brazil) to yield the CEb extract.

The biomass materials were lyophilized, pulverized, and then extracted with ethyl acetate and methanol 1:1 (*v*/*v*) added 1% formic acid by ultrasonic bath for 10 min. This methodology was previously optimized, and the results were better for the extraction (qualitative and quantitative). After the extraction, the mycelium was discarded, and the organic phase was concentrated by rotary evaporation (Buchi, São Paulo, Brazil) to yield the CEm extract.

### 2.4. Chemical Characterization of Compounds by HPLC-DAD-MS/MS

The chemical analyses were acquired on a UFLC-20AD Prominence Shimadzu coupled with a diode array detector (DAD) and mass spectrometer with electrospray ionization source and quadrupole and time-of-flight (MicrOTOF-Q III, Bruker Daltonics, Billerica, MA, USA) analyzers. A Kinetex^®^ C18 chromatography column (2.6 µm, 100 Å, 150 × 2.1 mm, Phenomenex, Torrance, CA, USA) was used. The flow rate was 0.3 mL/min, and the chromatographic column was maintained at 50 °C during the chromatographic analyses. The mobile phase was composed of acetonitrile (B) and water (A) with 0.1% (*v*/*v*) formic acid. The gradient elution profile was the following: 0–2 min (3% of B), 2–25 min (3–25% of B), 25–40 min (25–80% of B), and 40–43 min (80% of B, isocratic). For the MS analyses, nitrogen was applied as nebulizer (4 Bar), collision, and drying gas (9 L/min); the capillary voltage was 2500 kV. The samples were prepared at a concentration of 1 mg/mL in methanol and water (7:3, *v*/*v*), filtered on PTFE filters (Millex 0.22 mm × 13 mm, Millipore^®^, Burlington, MA, USA), and 2 μL of each sample were injected into the chromatographic system, except for the pool samples (quality control) that were injected with a volume of 5 µL. The pool was produced by mixing all samples from CEb and CEm. The UV analyses were monitored in the range 210–800 nm. The mass spectrometry data were acquired in positive and negative ion modes for all the samples.

### 2.5. Antibiofilm and Antibacterial Evaluations

Bacterial biofilm formation was quantified using a crystal violet assay on 96-well microtiter plates (Costar 3599, Corning, Inc., Corning, NY, USA) [14]. Into each well was pipetted 4 μL of each sample solubilized in DMSO, added to sterile water (76 μL), 40 μL of Brain Heart Infusion (BHI) broth for *Staphylococcus aureus* ATCC 6538 or Tryptone Soya Broth (TSB) for *Pseudomonas aeruginosa* ATCC 27853 and bacterial suspension (80 μL, ×10^8^ CFU/mL). Biofilm/bacterial growth control was performed with vehicle DMSO (4 μL) instead of the sample, and gentamicin 20 μg/mL (Sigma–Aldrich Co., St. Louis, MO, USA) was used as a control for the inhibition of bacterial growth. The samples were evaluated at concentrations of 20 and 200 μg/mL and the plates were incubated for 24 h at 37 °C. Subsequently, the planktonic cells were removed, and the wells were washed three times using sterile saline. Adherent biofilm was heat-fixed at 60 °C for 1 h, and then the biofilm biomass was stained with crystal violet (0.4% for 15 min at room temperature). Finally, the plate was washed with water, the biofilm was resuspended with ethanol for 30 min, and the absorbance (570 nm) was measured. The antibacterial activity was evaluated by the difference of final (t = 24 h) and initial bacterial growth (t = 0) using absorbance at 600 nm.

Minimum inhibitory concentration (MIC) was determined on 96-well microtiter plates as described above. The samples were serially diluted twofold in water and added to the wells, resulting in a final concentration ranging from 0.25 to 128 µg/mL. After 24 h of incubation at 37 °C, the MIC was defined as the lowest concentration of the sample with no visible bacterial growth in the well.

A protocol adapted from Zimmer et al. [15] was used to evaluate the eradication of *S. aureus* biofilm. At first, the biofilm was formed as aforementioned. After 24 h of incubation at 37 °C, planktonic cells were removed, and the samples were added (concentration range from 4 to 256 µg/mL). The plate was incubated again under the same conditions as before, and following this incubation the same steps used for the quantification of biofilm were performed. Biofilm formation controls were performed with DMSO (growth control) and vancomycin 20 μg/mL (Sigma–Aldrich Co., USA, activity control).

### 2.6. Processing Data and Statistical Analyses 

The raw data acquired by HPLC-DAD-MS were firstly visualized in DataAnalysis software (version 4.2, Bruker Daltonics GmbH, Bremen, Germany) and subsequently converted to .cdf. Next, the data were aligned by MetAlign software [16], producing a dataset with 5067 entries. These data were reduced by MSclust [17] which resulted in a dataset with 359 entries. The quality control sample was prepared from a mixture of 50 µL of each sample and it was injected every four samples during data acquisition. Subsequently, we manually refined the data using spreadsheets, and all entries up 5000 of peak intensities were considered. We also removed all the entries corresponding to the culture media constituents and the blank interferents. The statistical analyses were performed by the Metabolanalyst 5.0 platform [18]. The data from CEm and CEb were normalized by median, log-transformed, and data scaled by mean centering. We performed multivariate statistical analyses such as principal component analysis (PCA), volcano plot, hierarchical cluster analysis (HCA), partial least squares-discriminant analysis (PLS-DA), and heatmap. In addition, univariate analyses were also performed, such as Pattern-hunter, applying Pearson r, to determine the metabolites that correlated with the antibacterial activity against *S. aureus*. These biological results were added to the dataset after the application of the formula (100-bacterial growth percentage at 200 µg/mL × 100).

### 2.7. Molecular Networking and Data Analysis

The HPLC-MS data were converted to .mzXML format by MsConvert software [19]. The converted files were uploaded to the GNPS platform (http://gnps.ucsd.edu (accessed on 26 October 2022) [16] and classified into two groups of extract samples: CEb and CEm from *Penicillium* sp. 5MP2F4, and blank compounds (relative to extracts of culture media and solvent analyses) were removed. The molecular network was created using the online workflow on the GNPS platform (https://ccms-ucsd.github.io/GNPSDocumentation/ (accessed on 26 October 2022). The data were filtered by removing all MS/MS fragment ions within ±17 Da of the precursor ion. MS/MS spectra were window-filtered by selecting only the top six fragment ions in the ±50 Da window throughout the spectrum. For the precursor, ion mass tolerance was set to 0.02 Da, and for the MS/MS fragment ion tolerance was set to 0.08 Da. A molecular network was created where edges were filtered to have a cosine score above 0.6 and at least five matched peaks. The parameters for the library search were set to have a score above 0.6 and at least five matched peaks to assist in the metabolite annotation [20]. The molecular network job on GNPS can be found at https://gnps.ucsd.edu/ProteoSAFe/status.jsp?task=e513be1b31844e7ab44f504f7047bd9d (accessed on 26 October 2022) (positive mode). The results were exported and visualized in Cytoscape^®^ (version 3.9) [21].

### 2.8. Annotation of Compounds

The annotation of metabolites was performed based on the accurate mass spectral data to establish the molecular formula from the experimental high-resolution mass and isotope pattern, fragmentation pattern, and UV data in comparison with the obtained spectral data from literature databanks (such as MassBank, PubChem, NPAtlas, Louts, Chemical Abstract Service), and some compounds were confirmed by the injection of authentic standards. The molecular formula of each substance was determined considering errors and mSigma below 5 ppm and 30, respectively. Spectral comparisons were also performed by the Global Natural Products Social Molecular Networking (GNPS) platform.

## 3. Results

### 3.1. Identification of Fungal Strain

The isolated endophytic fungal strain 5MP2F4 was molecularly identified by the similarity with the ITS sequences deposited in the NCBI Blast tool using the Standard databases and the nucleotide collection (nr/nt). The search for similarity in the NCBI Blast tool was performed using the rRNA/ITS databases and the Internal transcribed spacer region (ITS) from fungi type. The sequence of the fungus 5MP2F4 showed 100% similarity with the sequence from GenBank code NR_111499, which belongs to the fungus identified as *P. brasilianum* [22]. In addition, several metabolites annotated here were also described for the species *P. brasilianum* [23]. Thus, the chemical data reinforced the possibility of the identification of strain 5MP2F4 as *P. brasilianum*.

### 3.2. OSMAC Experiments from Penicillium sp. 5MP2F4

The fungi 5MP2F4 was cultivated in different culture media (PDB, MEB, SAB, CZA, and YPD) in the OSMAC experiment, and it revealed visual changes in appearance, including the colors of the mycelia and broths (Figure S2). Additionally, the sporulation characteristics were also modified, which can be related to changes in the structure of the cell membrane formed [24]. The PDB medium showed high dark green sporulation and formation of light yellow exudates, differing from what was observed for the material cultured in CZA medium, where the mycelium was white and revealed no spores.

The fresh mycelial biomass from submerged cultures was freeze-dried and revealed a large amount of water (Figure S3). The lyophilized mycelia favored the extraction with the organic solvent to obtain the crude extracts from mycelia (CEm), since the presence of water can disrupt the obtention of CEm. The fresh to freeze-dried mass ratio reaches almost 12 in the biomass from the culture in MEB, and this ratio was around 5 to 7 times in the other culture media, while the extracts obtained from broths (CEb), cultivated in different media, were the highest mass yields in the cultivation with SAB and MEB (Figure S4).

### 3.3. Chemical Diversity of the Crude Extracts of Broth (CEb) and Mycelial Biomass (CEm) from Penicillium sp. Strain 5MP2F4 Obtained by OSMAC Strategy

**Figure 1:**
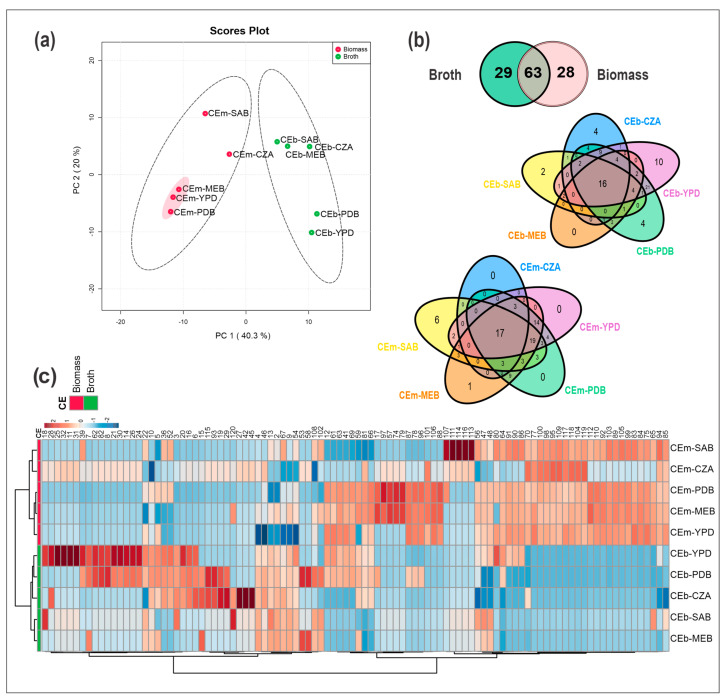
Metabolomic analyses from the crude extracts of mycelial biomass (CEm) and broth (CEb) of *Penicillium* sp. strain 5MP2F4 cultivated on different culture media (PDB, MEB, SAB, YPD, and CZA). (**a**) Principal component analysis (PCA) from all the crude extracts. The highlighted samples CEm-MEB, CEm-YPD, and CEm-PDB were the most active extracts against *S. aureus.* (**b**) Venn diagram of metabolites in CEb and CEm. (**c**) Heatmap and hierarchical clustering analysis (HCA) of the features ranging from red to blue color, representing high to low ion intensities. Groups of CEm and CEb samples are in red and green color, respectively.

To explore the chemodiversity of *Penicillium* sp. strain 5MP2F4, the OSMAC strategy together with metabolomics and chemometrics tools was applied to improve and broaden the chemical information from this strain. Then, the CEb and CEm extracts obtained from the OSMAC strategy were chemically profiled by HPLC-DAD-MS, along with all control samples to exclude their chromatographic peaks from the final datasets (Figures S5–S7). From the comparison between the CEb and CEm extracts, differences in their chemical profiles were observed (Figure 1). The CEb extracts showed mainly compounds with lower interactions to the C18 stationary phase used in the chromatographic analyses (retention times up to 32 min), while the CEm extracts revealed compounds with higher interactions (retention times above 31 min) (Figures S6 and S7).

The HPLC-MS data were initially aligned and clustered. The final dataset, used for subsequent chemometrics analyses, was obtained after the removal of control peaks and resulted in 120 features from the ten extracts (CEb and CEm). All spectral data for these compounds are summarized in Table S1, and the chromatographic peaks were numbered from 1 to 120 according to retention time and applied in the statistical analyses.

Multivariate statistical analyses were performed from the dataset of CEb and CEm. An unsupervised method of the chemical profiles was carried out by a principal component analysis (PCA), which revealed the formation of two groups constituted by the CEb and CEm extracts (Figure 1a). PC1 explained 40.3% of the data variance, while PC2 explained 20%, and both accounted for 60.3% of the total data variance.

From all the extracts analyzed in our study, the twenty-nine unique metabolites from CEb extracts were verified, while from CEm extracts there were twenty-eight unique metabolites (Figure 1b); moreover, they revealed significant ion intensity differences, as observed from the heatmap (Figure 1c). Regarding the number of metabolites biosynthesized in each culture medium, the compounds from CEb and CEm represented a total of 104 for the PDB medium, 97 for YPD, 63 for SAB, 78 for MEB, and 49 for CZA. These data can also be observed in Table S2, which summarizes the occurrence and diversity of metabolites from each extract.

Therefore, there are notable differences in the chemical profiles of broth and mycelial extracts from strain *Penicillium* sp. 5MP2F4. In addition, the variation in the culture media influenced the production of metabolites, some of which are dependent on the culture medium, such as the metabolite **32** that was found only from CEb-YPD. In contrast, the metabolite **120**, for example, was observed in the mycelial biomass and broth extracts from samples CEm-MEB, CEb-CZA, and CEb-SAB. These observations are useful, as they can aid in studies of targeted metabolites.

The heatmap and hierarchical cluster analysis (HCA) were constructed from the data of all CEb and CEm samples, and they are illustrated in Figure 1c. Variations in the occurrence of metabolites were also observed from the CEm and CEb extracts, as were the differences in ion intensities and diversity of compounds produced by the growth microorganism using the OSMAC strategy. In addition, the formation of two groups of samples, a group of CEm and another of CEb, was also detected in HCA, and their metabolites (with lower and higher retention times) were also observed in the heatmap. Thus, the CEb samples accumulated mainly compounds with lower retention times with the C18 stationary phase, while CEm were composed of metabolites with higher retention times.

The CEm-CZA and CEm-SAB extracts grouped in the same cluster, similar to what was observed in the PCA, where they clustered in the same quadrant; in contrast, CEm-YDB, CEm-MEB, and CEm-PDB clustered in another cluster belonging to the group of CEm extracts, demonstrating that these last three extracts show more chemical similarities. Interestingly, these similarities can also be observed in their antibacterial activities. For the CEb extracts, we observed similarities between the samples cultivated on culture media that differed from those observed for CEm. Thus, greater similarities were observed between CEb-MEB and CEb-SAB.

From the partial least squares discriminant analysis (PLS-DA), in which two relevant variables are (1) variable importance in projection (VIP) and (2) sum of the weights of the absolute regression coefficients, the graph with the VIP scores was obtained (Figure S8). The most relevant compounds for the separation of the CEm and CEb groups are mainly nonpolar (substances with higher numbering—high retention time) and polar metabolites (substances with lower numbering—low retention time), respectively. Thus, the compounds with the highest VIP scores and the top ten for CEm extracts were components **104** (palmitoyl-sn-glycerophosphatidylethanolamine), **100** (linoleoyl-sn-glycero-3-phosphocholine), **98** (linoleoyl-sn-glycero-3-phosphoethanolamine-isomer), **77**, **118**, **119** (octadecadienoic acid), **117**, **95** (linoleoyl-sn-glycero-3-phosphoethanolamine), **109** (lysophosphatidylethanolamine 18:1) and **103**, while for CEb they were **3** (unknown), **13**, **2** (5,6-dihydro-6-hydroxy penicillic acid), **9**, **16**, **4** (penicillic acid), **6** (orselinic acid), **10** (unknown), **20** (dihydroxyfumitremorgin C) and **7**. These appointments evidenced a wide chemical characterization from both samples of broths and mycelia.

### 3.4. Evaluation of Antibacterial and Antibiofilm Activities of Crude Extracts of Broth (CEb) and Mycelial Biomass (CEm) from Penicillium sp. Strain 5MP2F4 Obtained by OSMAC Strategy

To investigate further the antibacterial potential, all extracts (CEb and CEm) obtained by the strain *Penicillium* sp. 5MP2F4 were evaluated against *S. aureus* and *P. aeruginosa* to determine their activities on bacterial growth and biofilm formation.

**Figure 2:**
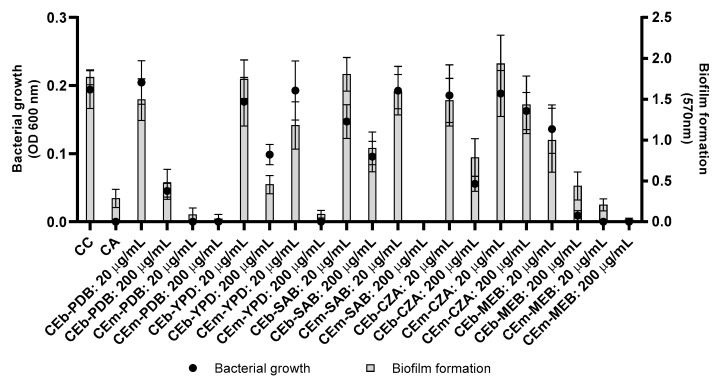
Biofilm formation and bacterial growth of *S. aureus* treated with the crude extracts of mycelial biomass (CEm—CEm-PDB, CEm-YPD, CEm-SAB, CEm-CZA, and CEm-MEB) and broth (CEb—CEb-PDB, CEb-YPD, CEb-SAB, CEb-CZA, and CEb-MEB) of *Penicillium* sp. strain 5MP2F4 at concentration 20 and 200 µg/mL. CC = growth control, culture medium only; CA = activity control, gentamicin 20 µg/mL.

The CEm and CEb extracts, at concentrations of 20 and 200 µg/mL, were initially evaluated against *S. aureus* and *P. aeruginosa*, but no inhibitory effect was observed against *P. aeruginosa* (Figure S9). The CEm and Ceb extracts revealed activity against *S. aureus* by inhibition of bacterial growth (Figure 2), except in the CEm-CZA extract. Subsequently, the CEm extracts were also evaluated against *S. aureus* at different concentrations (Figure S10). CEm-PDB (Figure S10a) and CEm-MEB (Figure S10e) were the most active and revealed MIC of 16 µg/mL, while the CEm-YPD (Figure S10b) and CEm-SAB (Figure S10c) extracts showed MIC of 64 and 128 µg/mL, respectively. The CEm-CZA (Figure S10d) extract was not active at the highest concentration tested (256 µg/mL).

The crude extracts from broth (CEb) were also evaluated in the assays of bacterial growth and biofilm formation against *S. aureus* at different concentrations (Figure S11), but these extracts showed moderate activity. The CEb-PDB (Figure S11a) inhibited more than 50% of the bacterial growth at 64 µg/mL. The CEb-MEB extract showed the best potential to inhibit bacterial growth, when compared to the CEb samples, resulting in an inhibition of approximately 50% of bacterial growth at the concentration of 32 µg/mL (Figure S11e).

Therefore, the CEb and CEm extracts presented classical antibiotic activity. In addition, the most promising antibacterial extracts, CEm-PDB and CEm-MEB, were also evaluated for the potential to eradicate the pre-formed bacterial biofilm (Figure S12). However, these extracts showed no potential to eradicate the biofilm.

### 3.5. Metabolomics and Statistical Analyses Applied to Determine the Chemical Differences between Samples with High and Moderate Antibacterial Activity

All CEb and CEm extracts were also evaluated for bacterial growth inhibition and revealed different activities against *S. aureus* (Figures S10a–e and S11a–e). From the MIC values of these samples, two groups were classified: (1) extracts with high activity, composed of CEm-PDB (MIC = 16 µg/mL), CEm-YPD (MIC = 64 µg/mL), and CEm-MEB (MIC = 16 µg/mL), (2) extracts with moderate activity, composed of CEm-SAB (MIC = 128 µg/mL), CEm-CZA (MIC ˃ 256 µg/mL), CEb-SAB (MIC ˃ 256 µg/mL), CEb-MEB (MIC ˃ 256 µg/mL), CEb-CZA (MIC ˃ 256 µg/mL), CEb-PDB (MIC ˃ 256 µg/mL) and CEb-YPD (MIC > 256 µg/mL). Thus, the statistical analyses were performed from chemical data and biological properties to propose the active metabolites.

**Figure 3:**
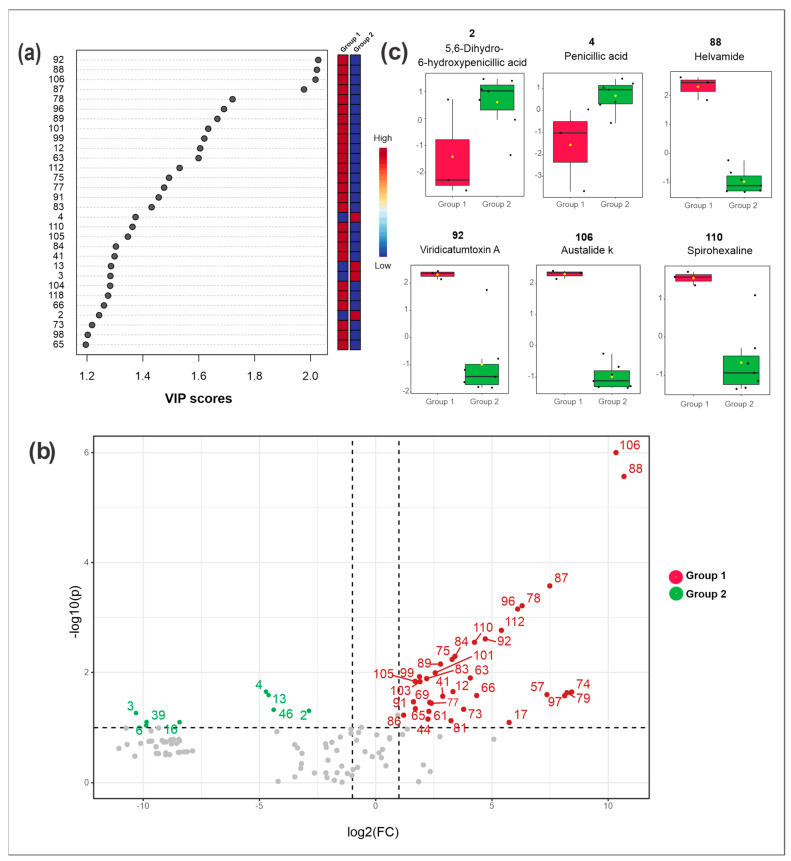
Metabolomics and statistical analyses from *Penicillium* sp. 5MP2F4. (**a**) Variable importance in projection (VIP) scores obtained by PLS-DA; (**b**) Volcano plot displaying the differences in metabolic profiles between the moderately active (represented in the left quadrant) and active (right quadrant) extracts. The x and y axes represent, respectively, the log2 of the quantitative variation of compounds (fold change, FC) and the −log10 of the p-value of all metabolites; (**c**) Box plots with the average distribution of ion intensities from the metabolites **2**, **4**, **88**, **92**, **106** and **110**. Group 1 (red): samples with higher antibacterial activity; Group 2 (green): samples with lower antibacterial activity; CEm: crude extract from mycelial biomass and CEb: crude extract from broth cultured in different media (PDB, MEB, CZA, and YPD).

The PCA analyses (Figure 1a) revealed more chemical similarities between the extracts with higher antibacterial activity (CEm-MEB, CEm-YPD, and CEm-PDB). From the Partial Least Squares Discriminant Analysis (PLS-DA), the variable importance in projection (VIP) with the sum of absolute regression coefficients (coef.) were plotted from 30 features (Figure 3a). Thus, the compounds **12**, **41**, **63**, **65–66**, **73**, **75**, **77–78**, **83–84**, **87–89**, **92**, **96**, **98–99**, **101**, **104–106**, **110**, **112**, and **118** were the most important for the separation of active samples. All the annotated metabolites are summarized in Table 1 and their spectral data in Table S1.

The Volcano plot (Figure 3b), which combines the fold change (FD) results and the statistical significance by t-test, revealed the statistically most relevant compounds for the separation of the samples of high (Group 1 in red) and moderate activities (Group 2 in green).

Compounds **2** (5,6-dihydroxy 6-hydroxy penicillic acid), **3**, **4** (penicillic acid), **6** (orselinic acid), **13**, **16**, **39** (spirotriprostatin G), and **46** were those statistically relevant for the samples of group 2 (moderate activity) compared to group 1 (high activity). The intensities of these compounds were higher in group 2 samples compared to group 1, as observed in the box plots for some of them (Figure 3c).

The metabolites marked in red (group 1) in Figure 3b are suggestively related to the biological activity and they are the main targets for the annotation, described in the subsequent section. Among these target compounds, we highlighted the following: **17** (brasiliamide E), **44** (austalide H acid), **63** (austalide Q acid), **66** (brasiliamide B), **73** (austalide J/13-*O*-desacetyl austalide), **75** (13-oxoverruculogen isomer), **78** (austalide P acid), **81** (chrysosporazine D), **87** (austalide L or W), **88** (helvamide), **92** (viridicatumtoxin A), **96** (austalide P), **101** (dihydroaustalide K), **106** (austalide k), **110** (spirohexaline) and **112** (pre-viridicatumtoxin). The box plots from some of them are illustrated in Figure 3c, and their highest intensities can be seen in those samples with the highest antibacterial activities.

These features, which were suggestively related to the biological activity, were also confirmed from biplots of PCA as strong variables for the most active extracts (cEm-PDB, cEm-YPB, and cEm-MEB) (Figure S13). Additionally, the compounds deoxy-fumitremorgin B (**89**), hydroxyfumitremorgin B (**90**), fumitrermorgin B and isomers (**86**, **91**, **94**), and di-deoxy-fumitremorgin B (**109**) were also visualized as strong variables for the CEm-PDB, CEm-YPD, and CEb-MEB.

### 3.6. Annotation of Constituents from Crude Extracts of Broth (cEb) and Mycelial Biomass (cEm) from Penicillium sp. Strain 5MP2F4 and Molecular Networking

The annotation of the constituents from cEb and cEm extracts is summarized in Table 1. The annotation was made from the spectral data of UV, high-resolution mass spectra, and fragmentation profile (Table S1) and compared to data described in the literature, and deposited in databanks, including the GNPS platform. In addition, some substances were confirmed by the injection of authentic standards. The MS and MS/MS data were also used to generate the molecular network and, thus, to organize the vast mass spectrometry datasets according to the similarity between fragmentation patterns (MS/MS) and their related precursor ions [25]. The compounds with similar fragmentation profiles were grouped into clusters that facilitated and expanded their annotation. We used the analyses of the CEb and CEm extracts, as well as the analyses of the controls (solvent/blank and culture media) to remove their peaks in the final analysis and for the construction of the molecular networking.

The molecular network is illustrated in Figure S14, where the thickness of the edges is proportional to the cosine values, and the entries with the intensity distributions receive green and yellow coloring relative to CEb and CEm, respectively. The overall molecular network visualization also indicated the differences between the CEb and CEm samples, highlighting the requirement of analyses from both materials (broth and mycelial biomass) to broaden the knowledge of microbial metabolite diversity from *Penicillium* sp. 5MP2F4.

**Figure 4:**
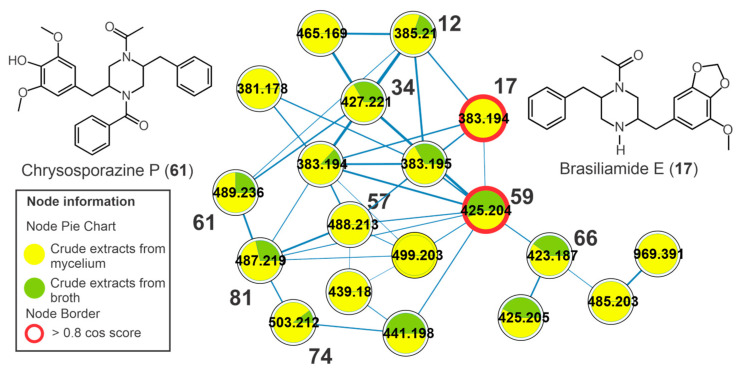
Cluster of brasiliamides from the molecular network of the crude extracts of mycelial biomass (CEm) and broth (CEb) of the endophytic fungi *Penicillium* sp. strain 5MP2F4, considering the positive ionization mode (ESI+) data. Nodes represent the parent ions, and edge strength indicates the chemical similarity between the MS/MS spectra. Groups of CEm and CEsob samples are in yellow and green coloring, respectively. The numbers represent the annotated metabolites which are described in Table 1, and the nodes circled in red indicate the compounds annotated by GNPS.

The chromatographic peaks **12** (*m/z* 385.2123 [M+H]^+^, C_22_H_29_N_2_O_4_^+^), **17** (*m/z* 383.1958 [M+H]^+^, C_22_H_27_N_2_O_4_^+^), **34** (*m/z* 427.2212 [M+H]^+^, C_24_H_31_N_2_O_5_^+^), **59** (*m/z* 425.2058 [M+H]^+^, C_24_H_29_N_2_O_5_^+^), **66** (*m/z* 423.1899 [M+H]+, C_24_H_27_N_2_O_5_^+^), **68** (*m/z* 439.1861 [M+H]+, C_24_H_27_N_2_O_6_^+^), and **72** (*m/z* 423.1865 [M+H]^+^, C_24_H_27_N_2_O_5_^+^) showed protonated ions and molecular formulas compatible with two nitrogen atoms (Table 1, Figure S15). These compounds were clustered together in the molecular network (Figure 4), and they revealed ketene losses (42 *u*) that indicated the presence of acetyl groups. From the fragmentation of the piperazine ring, it was possible to observe characteristic product ions at *m/z* 191 for compounds **17**, **59**, and **66**, which are relative to fragments containing the methoxylated aromatic ring and the methylenedioxy (Figure S16). For the other compounds, the modification of this fragment was important for the annotation of the compounds, and so **12**, **17**, **34**, **59**, **66**, **68,** and **72** (Figure S15) were annotated. This fragmentation profile is compatible with that observed in the spectra deposited at GNPS, and some features showed high cosine scores, such as brasiliamide D (**59**) and brasiliamide E (**17**). In the same cluster, two chrysosporazines, a rare class of phenylpropanoid piperazine alkaloid described for *Penicillium* species [26], were annotated, such as the metabolites **61** (*m/z* 489.2372 [M+H]^+^, C_29_H_33_N_2_O_5_^+^) and **81** (*m/z* 487.2217 [M+H]^+^, C_29_H_31_N_2_O_5_^+^) (Figure S15).

**Figure 5:**
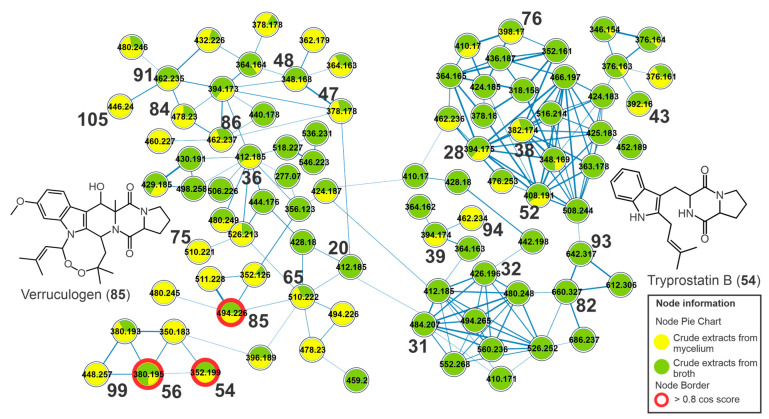
Cluster of diketopiperazine-type indolic alkaloids from the molecular network of the crude extracts of mycelial biomass (CEm) and broth (CEb) of the endophytic fungi *Penicillium* sp. Strain 5MP2F4, considering the positive ionization mode (ESI+) data. Nodes represent the parent ions, and edge strength indicates the chemical similarity between the MS/MS spectra. Groups of CEm and CEb samples are in yellow and green coloring, respectively. The numbers represent the annotated metabolites which are described in Table 1, and the nodes circled in red indicate the compounds annotated by GNPS.

A cluster yielded from indolic alkaloids of the diketopiperazine type and the metabolite verruculogen (**85**), for example, was observed in the molecular network (Figure 5). Compound **85** showed a UV spectrum similar to the chromophore of tryptophan [27] and an intense ion at *m/z* 494.2282 [M-H_2_O+H]^+^ (C_27_H_32_N_3_O_6_^+^), relative to the dehydration reaction in the ionization source. The fragment ion of **85** at *m/z* 352 (C_19_H_18_N_3_O_4_^+^) was yielded from the cleavage of the eight-membered ring. Subsequent cleavage of the piperazinodione ring produces the product ion at *m/z* 255.0766 (C_14_H_11_N_2_O_3_^+^) after the loss of C_5_H_7_NO and with subsequent loss of a CO molecule (28 *u*) producing the fragment at *m/z* 227.0808 [C_13_H_11_N_2_O_2_]^+^, similar to what was described in the literature [28]. This same fragmentation profile was observed for **83**, which exhibited a molecular formula containing additional oxygen compared to verruculogen (**85**). Thus, **83** was annotated as hydroxy verruculogen, with hydroxylation occurring on the eight-membered ring, and the rare substances 24-hydroxy verruculogen and 26-hydroxy verruculogen have been described in the literature from *Penicillium brefeldianum* [29]. Additionally, the metabolites **65**, **71,** and **75** revealed the same molecular formula, C_27_H_31_N_3_O_7_, a difference of 2 *u* in relation to verruculogen (**85**), and a similar fragmentation pathway – for example, the cleavage of endoperoxide with losses of 142 *u* and subsequent losses of water and carbon monoxide. The fragment ions at *m/z* 271, yielded from cleavage of the piperazinodione ring, were also observed. Thus, they were annotated as oxoverruculogen isomers (**65**, **71,** and **75**).

Peak **56** showed an intense ion at *m/z* 380.1947 [M+H]^+^, confirming the molecular formula C_22_H_25_N_3_O_3_. From this protonated ion, a loss of 56 *u* (C_4_H_8_) yielded the product ion at *m/z* 324.1337 (C_18_H_18_N_3_O_3_^+^), suggesting the 2-methylpropene group, and this undergoes a subsequent ring contraction with a loss of a CO molecule (28 *u*) and the generation of an ion at *m/z* 296.1374 (C_17_H_18_N_3_O_2_^+^). Cleavage in the piperazinodione ring by a loss of hexahydropyrrol [1,2-a]pyrazine-1,4-dione] yielded an intense product ion at *m/z* 226.1227 (C_15_H_16_NO^+^). An additional cleavage of the central ring resulted in the formation of the ion of *m/z* 212.105 (C_14_H_14_NO^+^), and the fragment ion *m/z* 255.1476 (C_16_H_19_N_2_O^+^) was yielded from the cleavage of the piperazinodione ring, and subsequently, the loss of 2-methylpropene yielded the ion at *m/z* 199.0868 (C_12_H_11_N_2_O^+^). This fragmentation profile is compatible with fumitremorgin C (**56**) described by Fornal et al. [28] and deposited at GNPS.

The compounds **20**, **22**, **36**, **37**, and **60** revealed the same molecular formula C_22_H_25_N_3_O_5_, which represents an addition of two oxygens compared to fumitremorgin C (**56**). They revealed losses of a water molecule (18 *u*) in the ionization source, indicating the presence of saturated hydroxyl groups; moreover, losses of 56 *u* (C_4_H_8_) with subsequent losses of CO and water molecules were also observed, such as the fragment ions at *m/z* 356 [M+H-C_4_H_8_]^+^, 338 [M+H-C_4_H_8_-H_2_O]^+^, and 310 [M+H-C_4_H_8_-H_2_O-CO]^+^ from **22**. Thus, they were annotated as dihydroxyfumitremorgin C/cyclotryprostatin A isomers.

The metabolite **94** showed an intense ion at *m/z* 462.2389 [M+H-H_2_O]^+^, compatible with the molecular formula C_27_H_33_N_3_O_5_, and relative to the dehydration reaction in the ionization source of the mass spectrometer. This intense dehydration reaction is common when there are OH substituents on the central ring from secondary and/or tertiary aliphatic alcohols, such as for the compounds **94** and **90**. The product ion of *m/z* 394, observed from *m/z* 462 (**94**), was yielded by the loss of an isoprenic unit (C_5_H_8_, 68 *u*), and subsequent elimination of the pyrrole ring produced the ion *m/z* 327. A similar fragmentation was observed for **86**, **89**–**91**, and **94,** which were annotated as fumitremorgin B isomers (**86**, **91**, **94**), deoxy-fumitremorgin B (**89**), and hydroxy-fumitremorgin B (**90**). Additionally, the compounds oxofumitremorgin B (**84**) and di-deoxy-fumitremorgin B (**99**) were also annotated. Several compounds annotated here are diketopiperazine-type indolic alkaloids, which have been described as precursors of the verruculogen biosynthetic pathway, as observed in Figure S17.

Compounds **54** and **64** revealed the same molecular formula, C_21_H_25_N_3_O_2_, and a similar fragmentation profile; moreover, **54** showed a score hit with tryprostatin B deposited in the GNPS. The product ions at *m/z* 296 [M+H-C_4_H_8_]^+^ and 268 [M+H-C_5_H_8_]^+^ indicated the presence of an isoprene group and yielded fragments with subsequent losses of CO (28 *u*) to produce the ions *m/z* 268 and 240. The peaks **54** and **64** were annotated as tryprostatin B (isomers). Moreover, the metabolites **26**, **28**, **30**, **38**, **39**, **40**, **43**, and **48** were annotated as dihydroxy-spirotryprostatin A, spirotryprostatin G, cyclotryprostatin E, cyclotryprostatin C or hydroxycycloprostatin A, cyclotryprostatin B, cclotryprostatin C, oxo-cyclotryprostatin A, and dehydroxycyclotryprostatin C, respectively.

**Figure 6:**
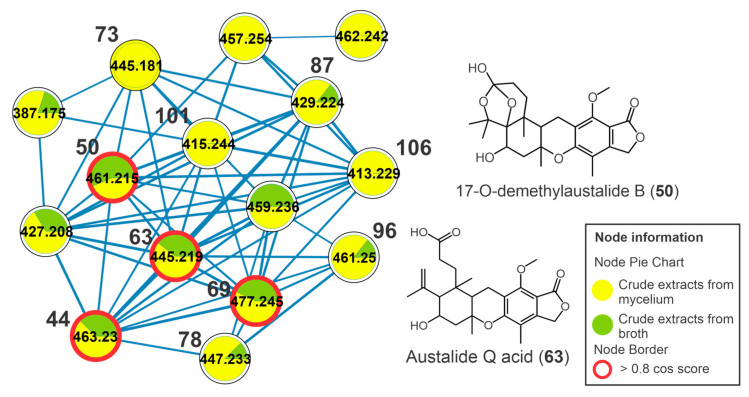
Cluster of austalides from the molecular network of the crude extracts of mycelial biomass (CEm) and broth (CEb) of the endophytic fungi *Penicillium* sp. strain 5MP2F4, considering the positive ionization mode (ESI+) data. Nodes represent parent ions, and edge strength indicates the chemical similarity between the MS/MS spectra. Groups of CEm and CEb samples are in yellow and green coloring, respectively. The numbers represent the annotated metabolites which are described in Table 1, and the nodes circled in red indicate the compounds annotated by GNPS.

It was possible to observe another cluster, formed by austalide-type meroterpenoids, which includes the compounds **44**, **50**, **63**, **69**, **73**, **78**, **87**, **96**, **101,** and **106** (Figure 6). These metabolites showed an absorption band at λ_max_ 270 nm in their UV spectra that is compatible with that described for austalides [30]. The compounds **44** (*m/z* 463.2310 [M+H]^+^, C_25_H_34_O_8_), **50** (*m/z* 461.2156 [M+H]^+^, C_25_H_32_O_8_), **63** (*m/z* 445.2216 [M+H]^+^, C_25_H_32_O_7_), **69** (*m/z* 477.2460 [M+H]^+^, C_26_H_36_O_8_), **73** (*m/z* 445.2198 [M+H]^+^, C_25_H_32_O_7_), **78** (*m/z* 447.2362 [M+H]^+^, C_25_H_34_O_7_), **87** (*m/z* 429.2259 [M+H]^+^, C_25_H_32_O_6_), **96** (*m/z* 443.2418 [M+H-H_2_O]^+^, C_26_H_36_O_7_), **101** (*m/z* 415.2484 [M+H]^+^, C_25_H_34_O_5_), and **106** (*m/z* 413.2400 [M+H]^+^, C_25_H_32_O_5_) (Table 1, Figure S18) revealed two diagnostic fragment ions at *m/z* 207 (C_11_H_11_O_4_^+^) and 177 (C_10_H_9_O_3_^+^). The fragment ion *m/z* 207 is yielded from retro Diels-Alder fission and subsequent loss of CH_2_O (30 *u*) that indicates the presence of the lactone group (Figure S19). For all the austalides, no modification was observed in the three rings fused to the benzolactone group. In addition, consecutive neutral losses of water molecules (18 *u*) indicated the presence of non-phenolic hydroxyls, such as those observed for the metabolites **44** (*m/z* 445 [M+H-H_2_O]^+^ and 409 [M+H-3xH_2_O]^+^) and **69** (*m/z* 459 [M+H-H_2_O]^+^ and 441 [M+H-2xH_2_O]^+^). Thus, these consecutive losses of water molecules were important in suggesting the aliphatic hydroxyl groups in the austalides, as well as the losses to yield the acylium fragment (e.g., losses of water or methanol molecules for acid or ester austalides, respectively).

The compound 17-*O*-desmethyl austalide B (**50**) was confirmed by the authentic standard. Beyond the similar fragmentation pathway described here, the metabolite **63** exhibited a cosine of 0.94 and 0.96 between the austalides **69** and **44**, revealing greater structural similarity between them. The metabolite **73** showed a cosine of 0.89 in relation to **87** and **101**. Thus, **63** and **73** were annotated as austalide Q acid and austalide J or 13-*O*-desacetyl austalide I, respectively. Metabolites **101** (*m/z* 415.2484 [M+H]^+^, C_25_H_34_O_5_) and **106** (*m/z* 413.2400 [M+H]^+^, C_25_H_32_O_5_) showed a fragmentation profile similar to that observed for austalides and the cosine between them was 0.97, suggesting a high similarity between them and a difference of 2 *u* relative to the presence of a double bond. They were annotated as dihydroaustalide K (**101**) and austalide K (**106**).

The chromatographic peaks **92**, **110,** and **112** showed two absorption bands, at λ_max_ ≈ 285 and 430 nm in their UV spectra. Their deprotonated ions *m/z* 564.1870, 563.1918, and 566.2027 are compatible with molecular formulas C_30_H_31_NO_10_, C_31_H_32_O_10_ e C_30_H_33_NO_10_, respectively. These compounds exhibited polyketide fragmentation patterns [31] with successive losses of water molecules (18 *u*), and for **92** and **112** an additional loss of an ammonia molecule (17 *u*) was also observed. Compound **92** was also confirmed by injection of authentic standards. Thus, **92**, **110,** and **112** were annotated as viridicatumtoxin A, spirohexalin, and previridicatum (Figure S20), respectively.

Lipid classes were also detected in the molecular network (Figure S21). A cluster generated from phosphate-free lipids was formed by the chromatographic peaks **111** (*m/z* 498.3711 [M+H]^+^, C_28_H_53_NO_6_^+^), **113** (*m/z* 474.375 [M+H]^+^, C_26_H_53_NO_6_^+^), **116** (*m/z* 500.3881 [M+H]^+^, C_28_H_55_NO_6_^+^), and **120** (*m/z* 760.6068 [M+H]+, C_46_H_82_NO_7_^+^). These compounds were annotated as isomers of lysodiacylglyceryltrimethylhomoserine (LDGTS) (**111**, **113**, **116**) and diacylglyceryl trimethylhomoserine (DGTS) (**120**). Additionally, a cluster with phospholipids was observed, and it was generated by compounds **95** (*m/z* 478.2869 [M+H]^+^, C_23_H_45_NO_7_P^+^), **98** (*m/z* 478.29 [M+H]^+^, C_23_H_45_NO_7_P^+^), **104** (*m/z* 454.2941 [M+H]^+^, C_21_H_45_NO_7_P^+^), **107** (*m/z* 498.3781 [M+H]^+^, C_27_H_53_N_3_O_3_P^+^), and **109** (*m/z* [M+H]^+^, C_23_H_47_NO_7_P^+^).

The compounds *N*-acetyl brasiliamide I (**34**), oxo-cyclotryprostatin A (**43**), chrysosporazine P (**61**), chrysosporazine D (**81**), helvamide (**88**), and oxoverrucologen isomers (**65**, **71**, and **75**) are described here for the first time in the genus *Penicillium*, while the metabolites viridicatumtoxin A (**92**), spirohexaline (**110**), previridicatumtoxin (**112**), austalide H acid (**44**), 17-*O*-desmethyl austalide B (**50**), austalide Q acid (**63**), austalide H (**69**), austalide J/13-*O*-desacety l austalide (**73**), austalide P acid (**78**), austalide L/W (**87**), austalide P (**96**), dihydroaustalide K (**101**), and austalide k (**106**) are described for the first time from a microorganism isolated in Brazil. Therefore, our study contributes to enlarging the chemical knowledge from the genus *Penicillium* and Brazilian microorganisms.

## 4. Discussion

Fungi can biosynthesize diverse structures with different polarities, functions, and biological properties (e.g., antimicrobial, immunomodulatory, antitumor, anti-inflammatory, antiviral, and antioxidant effects), and they can occur at different levels of abundance in each microorganism [3,6,25,31]. Consequently, in the research to describe the metabolic production capacity of a strain it is a challenge to perform a comprehensive screening with the detection of as many metabolites as possible. Routes of work aimed at isolating metabolites can often lead to known substances and a lot of work without innovation. Herein, an untargeted metabolomic approach from *Penicillium* sp. was applied to evaluate the effects of five different culture media on the metabolome of strain 5MP2F4.

This strain was collected as an endophyte from *Bromelia balansae* Mez that occurs in ironstone outcrops in the Pantanal biome of Mato Grosso do Sul, Brazil [32]. This plant species is adapted to several stressful environmental factors, such as conditions of a high incidence of UV radiation, shallow soils with few nutrients, and periods of water scarcity [33]. In addition to all these peculiar characteristics, studies on endophytes from these sites and from the Pantanal are scarce, especially those centered on the characterization of secondary metabolites [34,35,36,37,38].

The OSMAC strategy has been applied to obtain a greater diversity of microbial metabolites as well as in the search for new bioactive compounds [39]. Although OSMAC is a valuable and simple tool, several published studies apply it to guide metabolite isolation procedures; however, detailed information about chemical composition related to changes with different culture media is sparsely addressed, such as by more holistic appointments on chemicals by metabolomics tools and statistical analyses. In addition, the differences in metabolite production between extracts from broth and biomass of fungi are also scarcely reported by a wide approach, and several works used only mycelial biomass or broth fungal to obtain the crude extracts and to isolate the target metabolites [40,41,42].

Our efforts here involved the description of a great variability of metabolites produced by an endophytic fungus and the annotation of them from both broth and mycelial biomass, which revealed different chemical profiles. We also observed that the metabolites biosynthesized by strain *Penicillium* sp are dependent on the culture medium, since the exclusive metabolites were observed from a specific culture medium, as were differences in their intensities. These appointments have been described from strains of *Penicillium* with the application of the OSMAC strategy [41,42], but the authors used no wide chemical strategies to get information from these experiments, such as metabolomics.

From the statistical analyses illustrated in Figure 1, we highlighted the importance to study both the broth and the mycelial biomass, since they represented variations in the chemical profiles, resulting in significant changes in the antibacterial activity that opened up perspectives to find new bioactive compounds. A simple variation in the nutrients of the culture medium can lead to a great change in the production of metabolites and to enlarging the chemical diversity of a strain. For example, here the crude CEb-YPD and CEb-PDB extracts showed similar chemical compositions, while the crude mycelial biomass extracts, CEm-MEB and CEm-PDB, were the ones that showed more similarities.

The metabolomics data were also correlated with the antibacterial properties of the extracts (CEb and CEm obtained by OSMAC strategy) by statistical analyses to suggest the possible bioactive metabolites, optimizing the targets for further studies. The application of metabolomics and statistical tools to accelerate the research of bioactive compounds has been used by several studies, including compounds with antibacterial, larvicidal, and antileishmanial properties [25,43,44,45]. Thus, brasiliamides, austalides, diketopiperazine-type indolic alkaloids, and polyketides from the strain *Penicillium* sp. 5MP2F4 were highlighted here as chemical classes related to antibacterial activity against *S. aureus*.

The extracts from broth and biomass (CEb and CEm) showed different antibacterial activities and promising results to inhibit *S. aureus* bacterial growth. The increase in the number of bacterial infections together with the shortage of new antibiotics is a huge public health problem. It is estimated that the increase in the rates of bacterial infections caused by microorganisms ranks among the main causes of human mortality, and the bacteria *S. aureus* is on the list of multiresistant bacteria that call for the development of new drugs [46]. In this context, endophytic fungi have been highlighted as a relevant and important source to develop new drugs. They are poorly studied, since it is estimated that only 0.75–1.5% of the endophytes of plant species are known [47], highlighting the extensive goals to be studied [48].

In this work, we annotated different types of diketopiperazine-type alkaloids, such as brasiliamides A, C, D, E, I, and N-acetyl brasiliamide I. They are a class of metabolites widely distributed in fungi, bacteria, sponges, and actinomycetes. Brasiliamides are alkaloids that contain a piperazine grouping and they possess diverse biological activities including antibiotic, immunosuppressive, and antineoplastic activities, and they have great potential in future therapeutic applications [49]. Their biosynthesis has as an initial step the condensation of two or three amino acids as precursors [50], and they are considered rare in the literature, since only ten analogs have been described, such as brasiliamides A, B, C, D, E, F, G, H, I, J [50,51,52,53].

The brasiliamides A and B (Figure S15) were first described in 2002 from *Penicillium brasilianum* Batista JV-379, and their potential to produce convulsions in animals was evaluated, using the silkworm (*Bombys mori*) as an experimental model. Brasiliamide A and B showed ED50 of 300 and 50 µg/g, (concentration values of the substances per gram of diet), respectively. In this work, these brasiliamides were proposed as a new class of tremorgenic substances [52]. Later, this same research group described, for the first time, brasiliamides C, D and E from the cultivation of the same fungal strain. Brasiliamides C and D presented tremogenic potential with ED50 of 400 µg/g, while brasiliamide E was not active at the concentration of 500 µg/g [51].

Fill and co-workers described the isolation of brasiliamides A-F from *Penicillium brasilianum*, an endophyte of *Melia azedarach* (Meliaceae). The antimicrobial activity of brasiliamide A against a strain of *Bacillus subtilis* was also described, revealing an MIC of 250 µg/mL [53], and this substance was one of those found in our extracts. Furthermore, *P. brasilianum* WZXY-M122-9 was used to study the biosynthesis of brasiliamides and the implication of its enzymes. Thus, the authors described for the first time brasiliamide I [50], a metabolite also annotated here in our study. Additionally, *N*-acetyl brasiliamide I (**34**) was also annotated, which has not yet been reported in the literature.

Fumitrermorgin B (isomer) (**91**), oxoverruculogen (isomer) (**75**), hydroxy-verruculogen (**83**), deoxy-fumitremorgin B (**89**), di-deoxy-fumitremorgin B (**99**) are also compounds statically related to the antibacterial activity. These compounds are diketopiperazine-type indolic alkaloids that are commonly reported from *Aspergillus* and *Penicillium* species [54]. They have been related to different biological activities, such as antimicrobial, antiviral, anticancer, immunomodulatory, insecticidal, and antiphytopathogenic activities [55,56]. For this reason, these alkaloids are goals for drug development and their basic skeleton is an inspiration [54]. Diketopiperazine-type indolic alkaloids are biosynthesized by the condensation reaction of tryptophan with a second amino acid that can be a proline, phenylalanine, histidine, or leucine, yielding the indol diketopiperazine structure [54]. The metabolite fumitremorgin C (**56**) has been described as a potent inhibitor of breast cancer resistance protein [54,57], while verruculogen (**85**), a tremorgenic mycotoxin, acts in the central nervous system and induces sustained tremors, convulsions, and can lead to the death of animals. Furthermore, verruculogen inhibits the high conductance calcium-activated potassium channels of smooth muscle, and it has been a target of further drug research [58].

Austalides also presented correlations with antibacterial activity, including austalide Q acid (**63**), austalide P acid (**78**), austalide L/austalide W (**87**), austalide P (**96**), dihydroaustalide K (**101**), and austalide K (**106**). They are meroterpenoids with several activities described for them, such as antitumor, anti-inflammatory, antimicrobial, antiviral (influenza A H1N1), and osteoclast differentiation inhibitors [30]. Although few austalides have been evaluated against pathogenic bacteria, austalides M, N and R have been tested against sea-derived bacterial strains (*Halomonas aquamarina, Polaribacter irgensii, Pseudoalteromonas elyakovii, Roseobacter litoralis, Shewanella putrefaciens, Vibrio harveyi, V. natriegens* and *V. carchariae*), and they showed a broad spectrum of activity with MIC values between 0.001 and 1 µg/mL [59]. In addition, austalides M, N and R were also evaluated against the bacteria *S. aureus* and *Escherichia coli*, but they were not active [59]. These austalides were isolated from a strain of *Aspergillus* sp. isolated from the sponge *Tethya aurantium*, and they are suggested as antibacterial compounds useful in aquaculture [59].

The tetracycline-like polyketides, such as the annotated compounds viridicatumtoxin A (**92**), spirohexaline (**110**), and previridicatumtoxin (**112**) are recognized by their antibacterial potentials [60,61,62]. These fungal polyketides are an important class of metabolites with various biological activities described and other applications, such as pigmentation [63].

Viridicatumtoxin A (**92**) is a substance with a hybrid polyketide-isoprenoid structure. It is a rare tetracycline-like compound produced by fungi, which is structurally considered privileged because it possesses the ability to interact with different biological targets [64]. It presents potent antibacterial activity against different gram-positive strains, including some of clinical importance, for example, the strain of methicillin-resistant *S. aureus* with MIC of 0.5 µg/mL [62]. Viridicatumtoxin A has proven to be a useful potential antibiotic agent in times of increasing resistance; its total synthesis is described by Nicolaou [65], and its biosynthetic genes are also described. Thus, this tetracycline is a candidate for large-scale production, synthetically or genetically through BGC (biosynthetic gene cluster) modification for fermentation, and it is even used as a base skeleton to provide other relevant bioactive analogs [66].

## 5. Conclusions

From the endophytic fungi *Penicillium* sp. strain 5MP2F4, metabolomics, statistical, and molecular networking were applied and revealed broad chemical results from the crude extracts of broth (CEb) and mycelial biomass (CEm), including differences in the chemical profiles between them and their antibacterial activities. Several metabolites annotated here are reported for the first time from an endophytic fungus of Brazil and in the genus *Penicillium*. The OSMAC strategy induced the biosynthesis of different compounds and the application of metabolomics and chemometrics tools were useful in indicating antibacterial compounds, such as diketopiperazines, meroterpenoids, and polyketides, highlighting the rare tetracyclin viridicatumtoxin A. These findings reveal that the chemical and biological study of simple cultures can be useful in optimizing the knowledge of the metabolic potential of fungal strains.

## Figures and Tables

**Table 1:** Metabolites annotated from the crude extracts of *Penicillium* sp. 5MP2F4 by HPLC-DAD-MS/MS and their spectral data.

Peak	RT (min)	Compound	MF **	Positive Mode (*m/z*)
				[M+H]^+^
2	3.89	5,6-Dihydro-6-hydroxypenicillic acid	C_8_H_12_O_5_	189.0761
3	11.17	Unknown	C_11_H_16_O_7_S	275.0582 ^#^
4	11.68	Penicillic acid *	C_8_H_10_O_4_	171.0655
6	12.90	Orsellinic acid	C_8_H_8_O_4_	151.0384 ^#^
8	15.36	Dihydroxypropyl dihydroxy-methylbenzoate	C_11_H_14_O_6_	243.0866
10	16.33	Unknown	C_12_H_18_O_7_	275.1124
11	16.64	Unknown	C_10_H_11_NO_3_	194.0813
12	17.16	Brasiliamide I	C_22_H_28_N_2_O_4_	385.2123
17	21.74	Brasiliamide E	C_22_H_26_N_2_O_4_	383.1958
20	23.78	Dihydroxyfumitremorgin C or Cyclotryprostatin A	C_22_H_25_N_3_O_5_	412.1863
21	24.64	Unknown	C_22_H_25_N_3_O_7_	444.176
22	25.67	Dihydroxyfumitremorgin C or Cyclotryprostatin A	C_22_H_25_N_3_O_5_	412.1855
23	26.21	Triacetylfusarinin C	C_39_H_60_N_6_O_15_	853.4161
24	26.24	Unknown	C_24_H_25_N_7_O_3_	410.1701 ^F^
25	26.82	Unknown	C_22_H_25_N_3_O_7_	426.1664 ^#^
26	26.99	Dihydroxy-spirotryprostatin A	C_22_H_25_N_3_O_6_	410.1705 ^#^
28	27.11	Spirotryprostatin G	C_23_H_29_N_3_O_6_	394.1765
30	27.22	Cyclotryprostatin E	C_23_H_29_N_3_O_6_	412.1869 ^M^
31	27.36	Unknown	C_25_H_29_N_3_O_7_	484.2073
32	28.58	Unknown	C_22_H_23_N_3_O_6_	426.1963
33	28.66	Unknown	C_22_H_23_N_3_O_6_	410.1705 ^#^
34	28.76	N-acetyl brasiliamide I	C_24_H_30_N_2_O_5_	427.2212
36	29.10	Dihydroxyfumitremorgin C/Cyclotryprostatin A	C_22_H_25_N_3_O_5_	412.1861
37	29.38	Dihydroxyfumitremorgin C/Cyclotryprostatin A	C_22_H_25_N_3_O_5_	394.1752 ^#^
38	29.76	Cyclotryprostatin C /hydroxycycloprostatin A	C_21_H_23_N_3_O_4_	382.1755
39	30.08	Cyclotryprostatin B	C_23_H_27_N_3_O_5_	394.1756
40	30.14	Cyclotryprostatin C	C_21_H_21_N_3_O_3_	364.1561
41	30.16	Unknown	C_30_H_34_N_6_O_4_	555.2699
43	30.62	Oxo-cyclotryprostatin A	C_22_H_23_N_3_O_5_	392.1600 ^#^
45	30.66	Abscisic acid	C_15_H_20_O_4_	265.1439
44	30.66	Austalide H acid	C_25_H_34_O_8_	463.231
47	30.97	Unknown	C_22_H_25_N_3_O_4_	378.179 ^#^
48	31.01	Dehydroxycyclotryprostatin C	C_21_H_23_N_3_O_3_	348.1704 ^#^
50	31.23	17-*O*-desmethyl austalide B *	C_25_H_32_O_8_	461.2156
52	31.32	Unknown	C_23_H_25_N_3_O_4_	408.1893
53	31.46	Unknown	C_22_H_14_N_4_O_8_	463.087
54	31.49	Tryprostatin B	C_21_H_25_N_3_O_2_	352.2019
55	31.74	Cyclodepsipeptide (JBIR-113)	C_31_H_41_N_5_O_7_	596.3086
56	31.89	Fumitremorgin C	C_22_H_25_N_3_O_3_	380.1947
57	32.08	Unknown	C_28_H_29_N_3_O_5_	488.2171
58	32.13	Unknown	C_21_H_18_O_12_	431.0599 ^M^
59	32.14	Brasiliamide D	C_24_H_28_N_2_O_5_	425.2058
60	32.37	Dihydroxyfumitremorgin C/Cyclotryprostatin A	C_22_H_25_N_3_O_5_	412.1854
61	32.43	Chrysosporazine P	C_29_H_32_N_2_O_5_	489.2372
63	32.48	Austalide Q acid	C_25_H_32_O_7_	445.2216
64	32.69	Tryprostatin B isomer	C_21_H_25_N_3_O_2_	352.2014
65	32.82	Oxoverrucologen (isomer)	C_27_H_31_N_3_O_7_	510.222
66	32.90	Brasiliamide C	C_24_H_26_N_2_O_5_	423.1899
68	33.08	Brasiliamide A	C_24_H_26_N_2_O_6_	439.1861
69	33.19	Austalide H	C_26_H_36_O_8_	477.2460
70	33.29	Unknown	C_33_H_36_N_2_O_3_	509.2747
71	33.34	Oxoverruculogen	C_27_H_31_N_3_O_7_	510.2316
72	33.50	Brasiliamide B	C_24_H_26_N_2_O_5_	423.1865
73	33.58	13-*O*-deacetyl austalide I/austalide J	C_25_H_32_O_7_	445.2198
74	33.91	Unknown	C_29_H_30_N_2_O_6_	503.2177
75	34.07	Oxoverruculogen (isomer)	C_27_H_31_N_3_O_7_	510.2231
76	34.45	Indole alkaloid—diketopiperazine	C_21_H_23_N_3_O_5_	398.1712
78	34.61	Austalide P acid	C_25_H_34_O_7_	447.2362
80	34.68	Oxo-Hydroxyfumitremorgin C	C_22_H_23_N_3_O_5_	410.1712
81	34.74	Chrysosporazine D	C_29_H_30_N_2_O_5_	487.2217
82	34.84	Unknown	C_37_H_46_N_3_O_8_	660.329
83	35.09	Hydroxy-verruculogen	C_27_H_33_N_3_O_8_	510.2213 ^#^
84	35.09	Oxofumitremorgin B	C_27_H_31_N_3_O_5_	478.2324
85	35.39	Verruculogen	C_27_H_33_N_3_O_7_	494.2282 ^#^
86	35.59	Fumitrermorgin B (isomer)	C_27_H_33_N_3_O_5_	462.2375 ^#^
87	35.82	Austalide L/Austalide W	C_25_H_32_O_6_	429.2259
88	35.94	Helvamide	C_29_H_28_N_2_O_5_	485.2076
89	36.21	Deoxy-fumitremorgin B	C_27_H_33_N_3_O_4_	446.2426 ^#^
90	36.35	Hydroxyfumitremorgin B	C_27_H_33_N_3_O_6_	478.2325 ^#^
91	36.41	Fumitrermorgin B (isomer)	C_27_H_33_N_3_O_5_	462.2375 ^#^
92	36.55	Viridicatumtoxin A *	C_30_H_31_NO_10_	548.1909 ^#^
93	36.55	Indole alkaloid	C_37_H_43_N_3_O_7_	642.3179
94	36.58	Fumitrermorgin B	C_27_H_33_N_3_O_5_	462.2389 ^#^
95	36.68	Linoleoyl-sn-glycero-3-phosphoethanolamine (isomer)	C_23_H_44_NO_7_P	478.2869
96	36.78	Austalide P	C_26_H_36_O_7_	443.2418 ^#^
97	36.83	Viridicatum toxin A (isomer)	C_30_H_31_NO_10_	548.1909 ^#^
98	37.02	Linoleoyl-sn-glycero-3-phosphoethanolamine (isomer)	C_23_H_44_NO_7_P	478.29
99	37.05	Di-deoxy-fumitremorgin B	C_27_H_33_N_3_O_3_	448.2574
100	37.26	Linoleoyl-sn-glycero-3-phosphocholine	C_26_H_50_NO_7_P	520.3381
101	37.33	Dihydroaustalide K	C_25_H_34_O_5_	415.2484
102	37.43	GameXPeptide C	C_28_H_57_NO_9_	552.4114
104	37.75	Palmitoyl-sn-glycerophosphatidylethanolamine	C_21_H_44_NO_7_P	454.2941
105	37.93	Indole alkaloid—diketopiperazine	C_27_H_31_N_3_O_3_	446.2434
106	38.04	Austalide K	C_25_H_32_O_5_	413.24
107	38.07	Unknown	C_27_H_52_N_3_O_3_P	498.3781
108	38.13	GameXPeptide D	C_30_H_55_N_5_O_5_	566.4266
109	38.29	Lysophosphatidylethanolamine (18:1)	C_23_H_47_NO_7_P	480.3082
110	38.40	Spirohexaline	C_31_H_32_O_10_	547.1949 ^#^
111	38.46	LDGTS 18:2	C_28_H_52_NO_6_	498.3711
112	38.73	Previridicatumtoxin	C_30_H_33_NO_10_	550.2062 ^#^
113	39.36	LDGTS 16:0	C_26_H_52_NO_6_	474.375
116	39.86	LDGTS 18:1	C_28_H_54_NO_6_	500.3881
119	42.29	Octadecadienoic acid	C_18_H_32_O_2_	303.2282 ^N^
120	42.30	DGTS 18:2-18:2	C_46_H_81_NO_7_	760.6068

MF: Molecular Formula; RT: retention time; *: confirmed by an authentic standard; ** determined by considering errors and mSigma less than 8 ppm and 30, respectively. ^#^: [M+H-H_2_O]^+^, ^N^: [M+Na]^+^, ^F^: [M+H-HCOOH]^+, M^: [M+H-CH_3_OH]^+^.

## Data Availability

All data are available in this publication.

## Supplementary Materials

The following supporting information can be downloaded at: https://www.mdpi.com/article/10.3390/metabo13020236/s1, Figure S1. *Penicillium* sp. strain 5MP2F4 at 7-day cultured in PDA, photo of front (A) and reverse (B) of culture. Figure S2. Cultivation of *Penicillium* sp. strain 5MP2F4 in different nutrient conditions. A: PDB (Potato Dextrose Broth); B: MEB (Malt Extract Broth); C: SAB (Sabouraud); D: CZA (Czapek); E: YPD (Yeast extract Peptone Dextrose). Figure S3. Fresh and freeze-dried biomass obtained from the growth of *Penicillium* sp. strain 5MP2F4 on different types of culture medium. Figure S4. Yields of crude extracts obtained from the broth of *Penicillium* sp. strain 5MP2F4 (CEb) in different nutrient conditions PDB (Potato Dextrose Broth), MEB (Malt Extract Broth), SAB (Sabouraud), CZA (Czapek), and YPD (Yeast extract Peptone Dextrose). Figure S5. Base peak chromatograms corresponding to crude extracts from different types of medium blanks used in OSMAC experiment obtained in positive ion mode by HPLC-MS. Figure S6. Base peak chromatograms obtained in positive ionization mode by HPLC-MS of the crude extracts of broth (CEb) from *Penicillium* sp. strain 5MP2F4 cultures on different types of medium. Figure S7. Base peak chromatograms obtained in positive ionization mode by HPLC-MS of the crude extracts of biomass (CEm) from *Penicillium* sp. strain 5MP2F4 cultures on different types of medium. Figure S8. Metabolomic study of the *Penicillium* sp. strain 5MP2F4 in relation to different extracts from broth (CEb-PDB, CEb-MEB, CEb-SAB, CEb-YPD, and CEb-CZA) and mycelial biomass (CEm-PDB, CEm-MEB, CEm-SAB, CEm-YPD, and CEm-CZA). Major metabolites identified by PLS-DA and VIP score analyses on the x-axis. The colored squares on the right indicate the relative intensities of the compounds in each group. Figure S9. Biofilm formation and bacterial growth of *P. aeruginosa* (ATCC 27853) in the presence of 20 and 200 µg/mL of the broth (CEb) and biomass crude extracts (CEb) from *Penicillium* sp. strain 5MP2F4. CC = Growth control, culture medium only; CA = Activity control, gentamicin 20 µg/mL. Figure S10. Biofilm formation and bacterial growth of *S. aureus* (ATCC 6538) treated with the biomass crude extracts of *Penicillium* sp. strain 5MP2F4 ((a) CEm-PDB; (b) CEm-YPD; (c) CEm-SAB; (d) CEm-CZA; (e) CEm-MEB) at different concentrations. CC = Growth control, culture medium only; CA = activity control, gentamicin 20 µg/mL. Figure S11. Biofilm formation and bacterial growth of *S. aureus* treated with broth crude extracts of *Penicillium* sp. strain 5MP2F4. (a) CEb-PDB; (b) CEb-YPD; (c) CEb-SAB; (d) CEb-CZA; (e) CEb-MEB) at different concentrations. CC = Growth control, culture medium only; CA = activity control, gentamicin 20 µg/mL. Figure S12. Pre-formed biofilm *S. aureus* treated with the CEm-PDB and CEm-MEB biomass extracts of *Penicillium* sp. strain 5MP2F4 at different concentrations. CC = Growth control; CA = activity control, vancomycin at 20 µg/mL. Figure S13. Biplot of principal component 1 (PC1) and principal component 2 (PC2). The samples CEm-PDB, CEm-YPD, and CE-MEB are the most active against *S. aureus*. Figure S14. Molecular networking of crude extracts from *Penicillium* sp. strain 5MP2F4, using positive ionization mode (ESI+) data. Crude extracts from broth (CEb) samples are colored green and Crude extracts from biomass (CEm) samples are colored yellow. The edge strength is proportional to the cosine values. Figure S15. Structures of brasiliamides and chrysosporazines annotated in extracts of *Penicillium* sp. strain 5MP2F4. Figure S16. Fragmentation proposal for some observed ions for metabolite 59. Figure S17. Simplified proposal of the biosynthetic pathway for verruculogen based on Han et al. [67]. Metabolites with the indication of numbers were annotated from extracts of *Penicillium* sp. strain 5MP2F4. Figure S18. Structures of austalide-type meroterpenoid annotated from extracts of *Penicillium* sp. strain 5MP2F4. Figure S19. Proposed fragmentation pathway for metabolite 44. Figure S20. Polyketides annotated from extracts of *Penicillium* sp. strain 5MP2F4. Figure S21. Cluster of lipid compounds from the molecular network of the crude extracts of mycelium biomass (CEm) and broth (CEb) of the endophytic fungi *Penicillium* sp. strain 5MP2F4, considering the positive ionization mode (ESI+) data. Nodes represent parent ions, and edge strength indicates the chemical similarity between the MS/MS spectra. Groups of CEm and CEb samples are in yellow and green color, respectively. The numbers represent the annotated metabolites described in Table 1, and the nodes circled in red indicate the compounds annotated by GNPS. Table S1: Table of spectral data of constituents of biomass and broth crude extracts from *Penicillium* sp. strain 5MP2F4 by HPLC-DAD-MS/MS. Table S2. Presence or absence of compounds in the extracts of broth and biomass of *Penicillium* sp. strain 5MP2F4 by HPLC-MS analysis.
