# Supplementary material for: Metabolomic Strategies to Improve Chemical Information from OSMAC Studies of Endophytic Fungi
Source: Metabolites. 2023 Feb 5;13(2):236. doi: 10.3390/metabo13020236 (PMC9961420; doi:10.3390/metabo13020236)
Supplement: Supplementary file 1 [file metabolites-13-00236-s001.zip › metabolites-2178323-supplementary.pdf]

metabolites

MDPI

Supplementary Material

Metabolomic Strategies to Improve the Chemical Information from OSMAC Studies of Endophytic Fungi

Fernanda Motta Ribeiro da Silva [1], Gecele Matos Paggi [2], Flávia Roberta Brust [3], Alexandre José Macedo [3] and Denise Brentan Silva [1,*]

[1] Laboratory of Natural Products and Mass Spectrometry (LaPNEM), Federal University of Mato Grosso do Sul, Campo Grande 79070-900, Brazil
[2] Laboratory of Ecology and Evolutionary Biology (LEBio), Institute of Biosciences, Federal University of Mato Grosso do Sul, Campo Grande 79070-900, Brazil
[3] Biofilms and Diversity Laboratory, Faculty of Pharmacy and Biotechnology Center, Federal University of Rio Grande do Sul, Porto Alegre 91501-970, Brazil
* Correspondence: denise.brentan@ufms.br

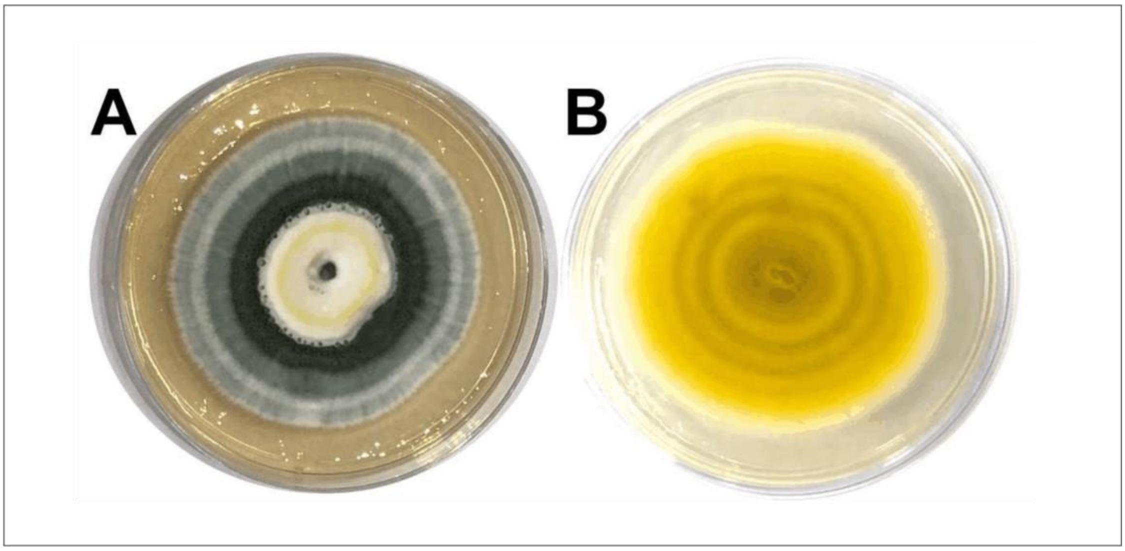

Figure S1. *Penicillium* sp. strain 5MP2F4 at 7-day cultured in PDA, photo of front (A) and reverse (B) of culture.

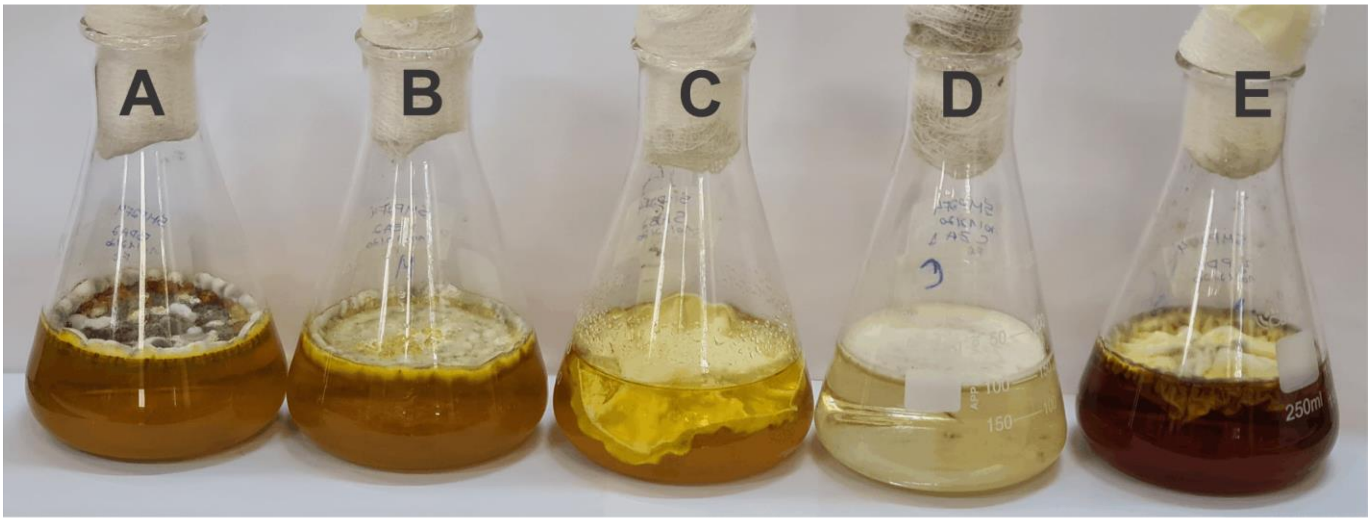

Figure S2. Cultivation of *Penicillium* sp. strain 5MP2F4 in different nutrient conditions. A: PDB (Potato Dextrose Broth); B: MEB (Malt Extract Broth); C: SAB (Sabouraud); D: CZA (Czapek); E: YPD (Yeast extract Peptone Dextrose).

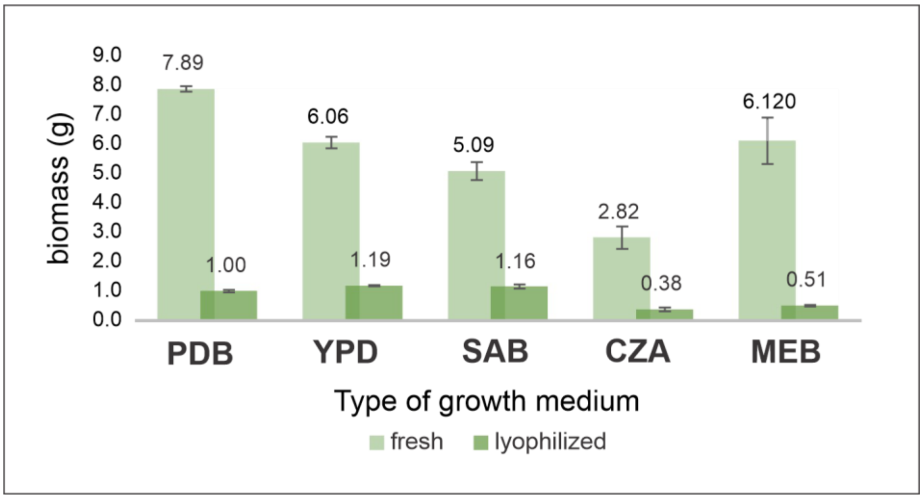

Figure S3. Fresh and freeze-dried biomass obtained from the growth of *Penicillium* sp. strain 5MP2F4 on different types of culture medium.

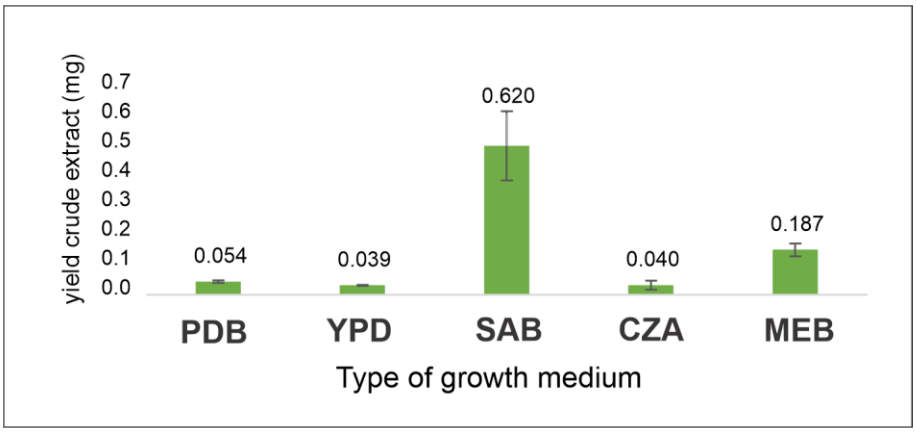

Figure S4 - Yields of crude extracts obtained from the broth of *Penicillium* sp. strain 5MP2F4 (CEb) in different nutrient conditions PDB (Potato Dextrose Broth), MEB (Malt Extract Broth), SAB (Sabouraud), CZA (Czapek), and YPD (Yeast extract Peptone Dextrose).

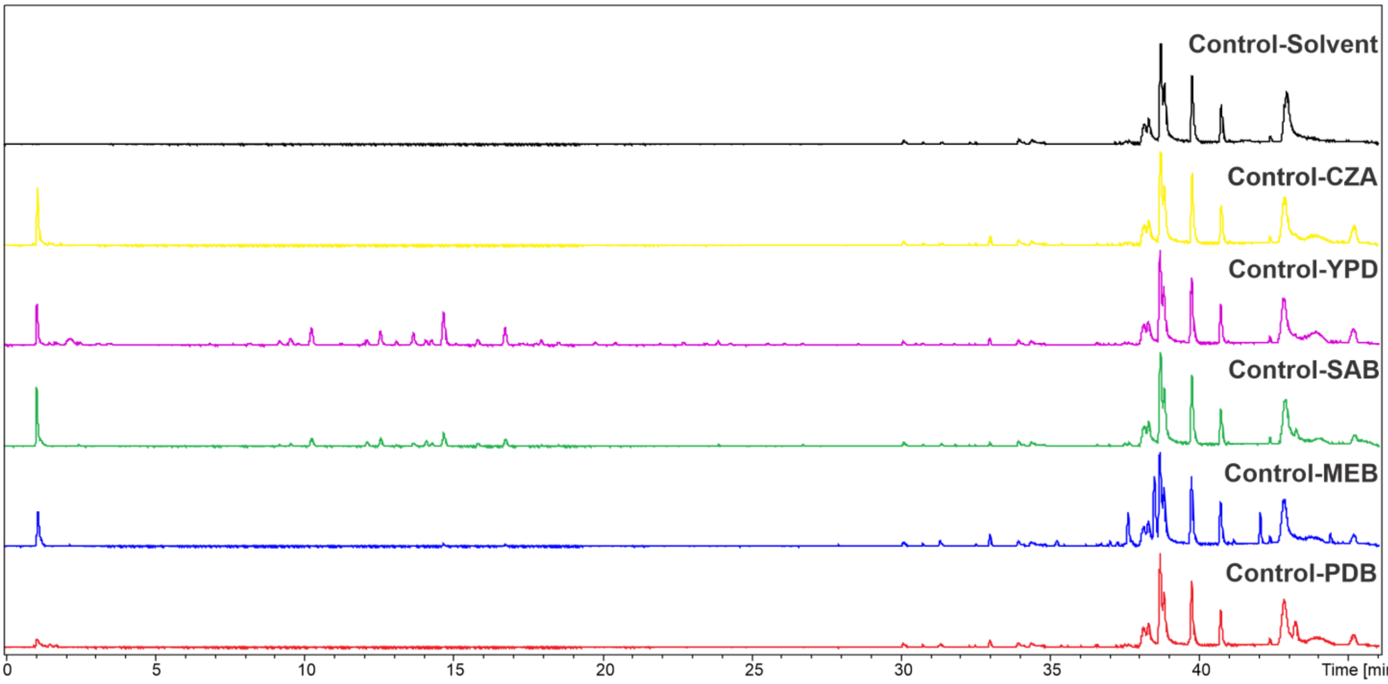

Figure S5. Base peak chromatograms corresponding to crude extracts from different types of medium blanks used in OSMAC experiment obtained in positive ion mode by HPLC-MS.

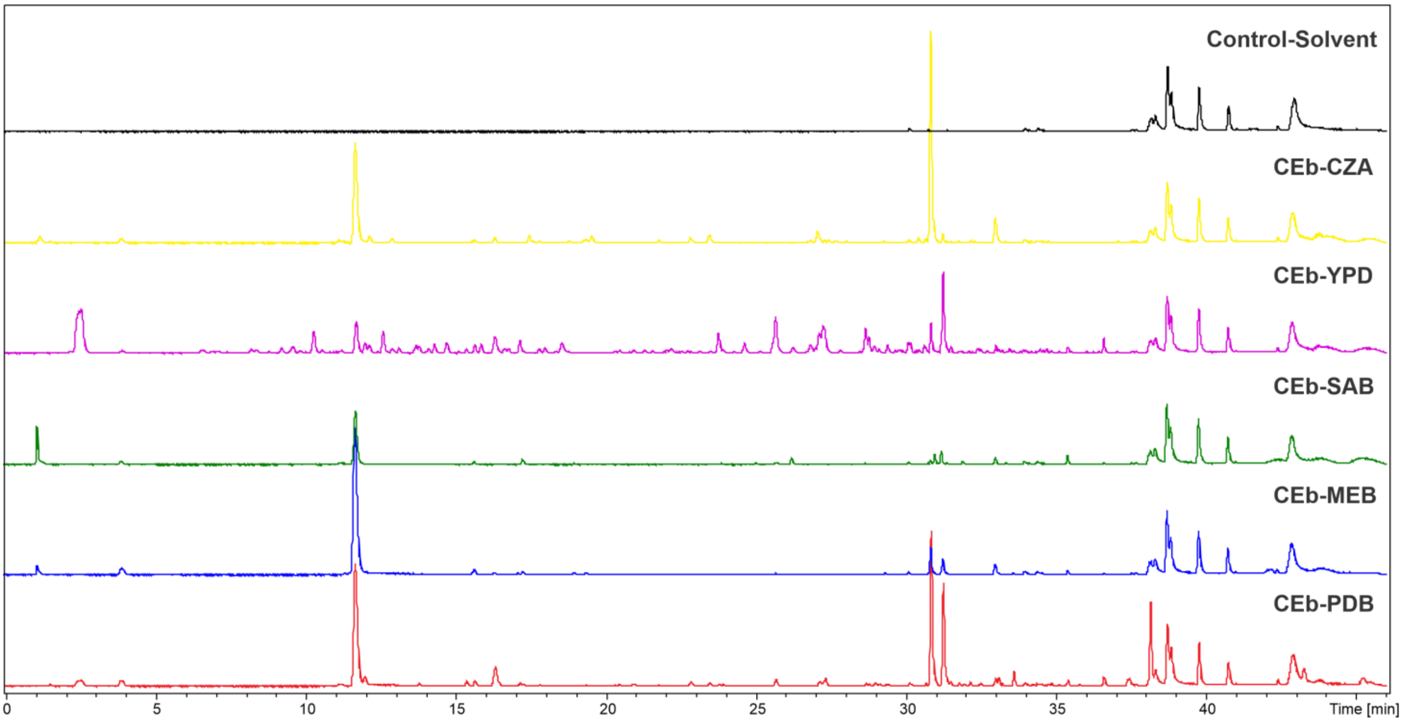

Figure S6. Base peak chromatograms obtained in positive ionization mode by HPLC-MS of the crude extracts of broth (CEb) from *Penicillium* sp. strain 5MP2F4 cultures on different types of medium.

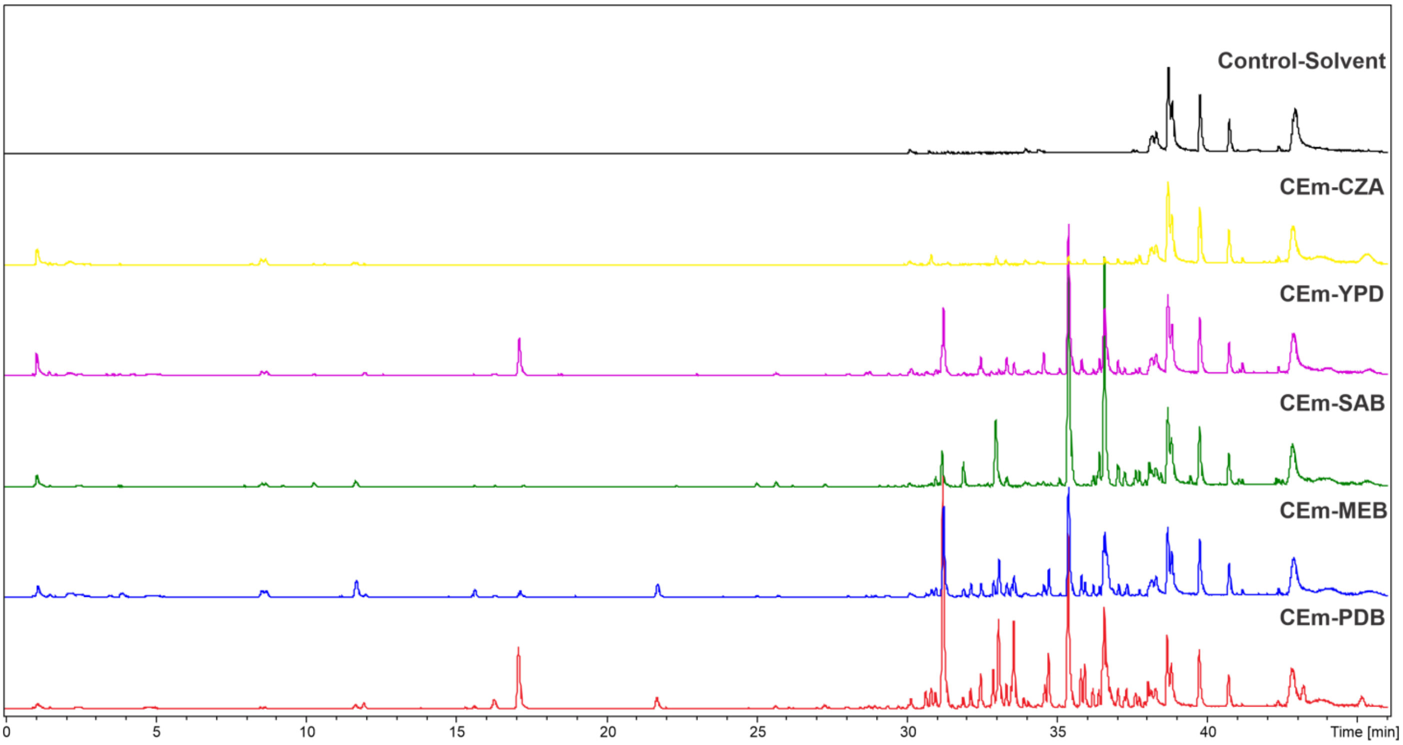

Figure S7. Base peak chromatograms obtained in positive ionization mode by HPLC-MS of the crude extracts of biomass (CEm) from *Penicillium* sp. strain 5MP2F4 cultures on different types of medium.

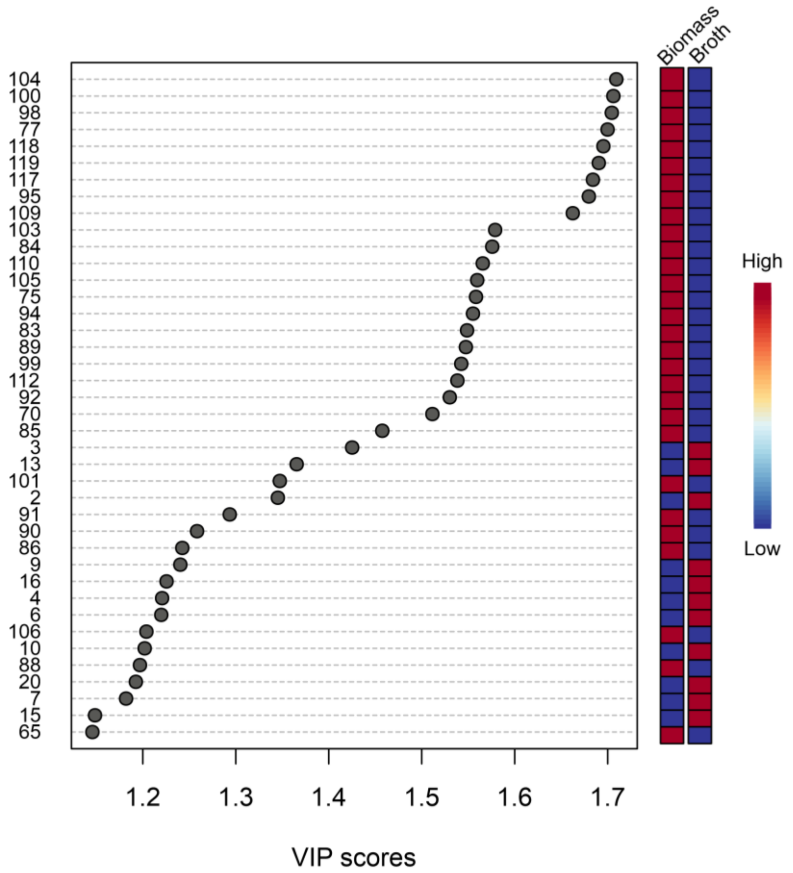

Figure S8. Metabolomic study of the *Penicillium* sp. strain 5MP2F4 in relation to different extracts from broth (CEb-PDB, CEb-MEB, CEb-SAB, CE-YPD, and CEb-CZA) and mycelial biomass (CEm-PDB, CEm-MEB, CEm-SAB, CEm-YPD, and CEm-CZA). Major metabolites identified by PLS-DA and VIP score analyses on the x-axis. The colored squares on the right indicate the relative intensities of the compounds in each group.

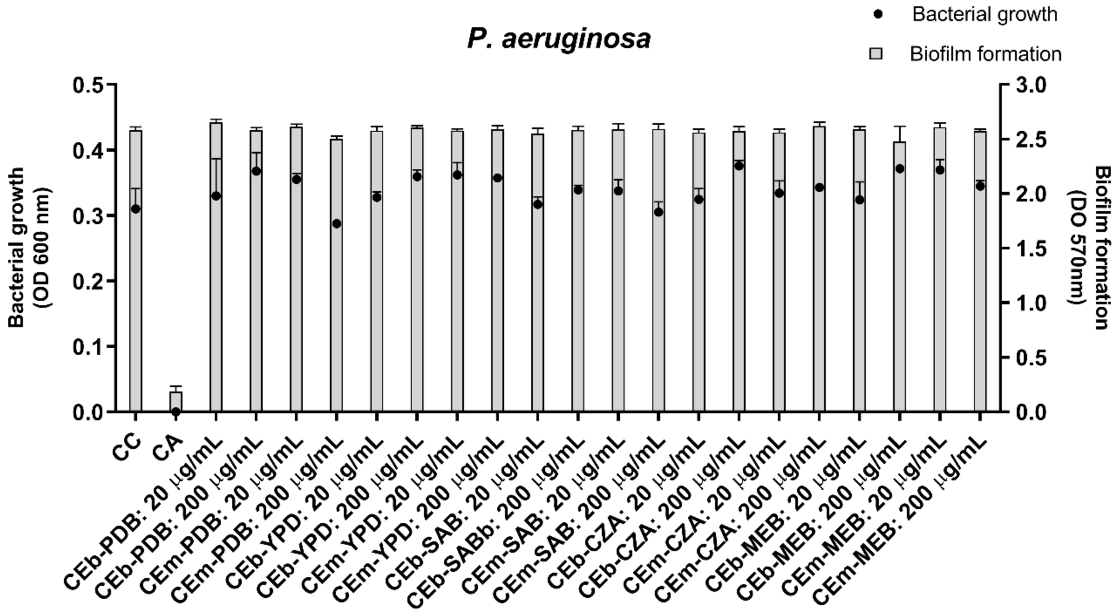

Figure S9 - Biofilm formation and bacterial growth of *P. aeruginosa* (ATCC 27853) in the presence of 20 and 200 µg/mL of the broth (CEb) and biomass crude extracts (CEb) from *Penicillium* sp. strain 5MP2F4. CC = Growth control, culture medium only; CA = Activity control, gentamicin 20 µg/mL

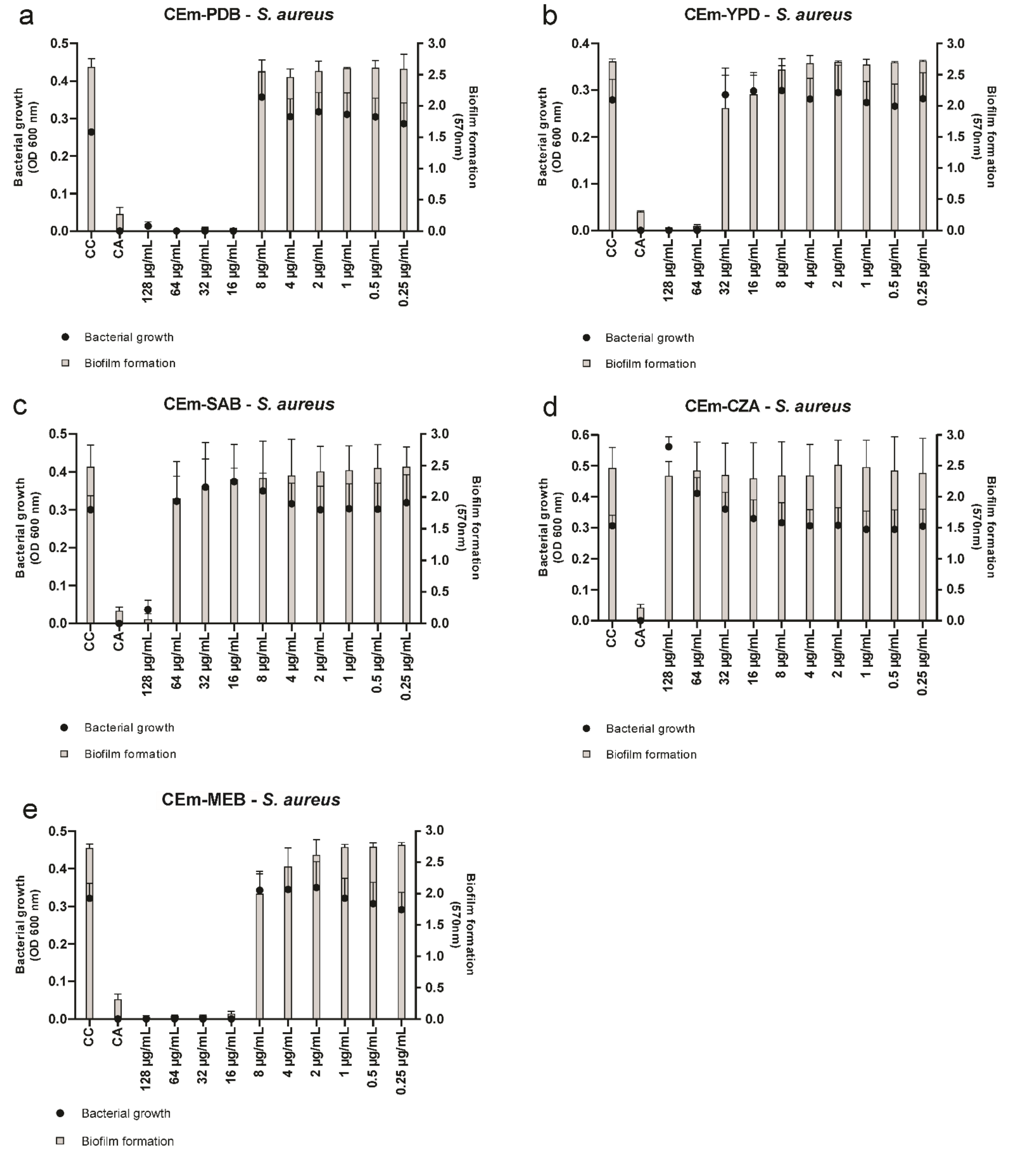

Figure S10. Biofilm formation and bacterial growth of *S. aureus* (ATCC 6538) treated with the biomass crude extracts of *Penicillium* sp. strain 5MP2F4 ((a) CEm-PDB; (b) CEm-YPD; (c) CEm-SAB; (d) CEm-CZA; (e) CEm-MEB) at different concentrations. CC = Growth control, culture medium only; CA = activity control, gentamicin 20 µg/mL.

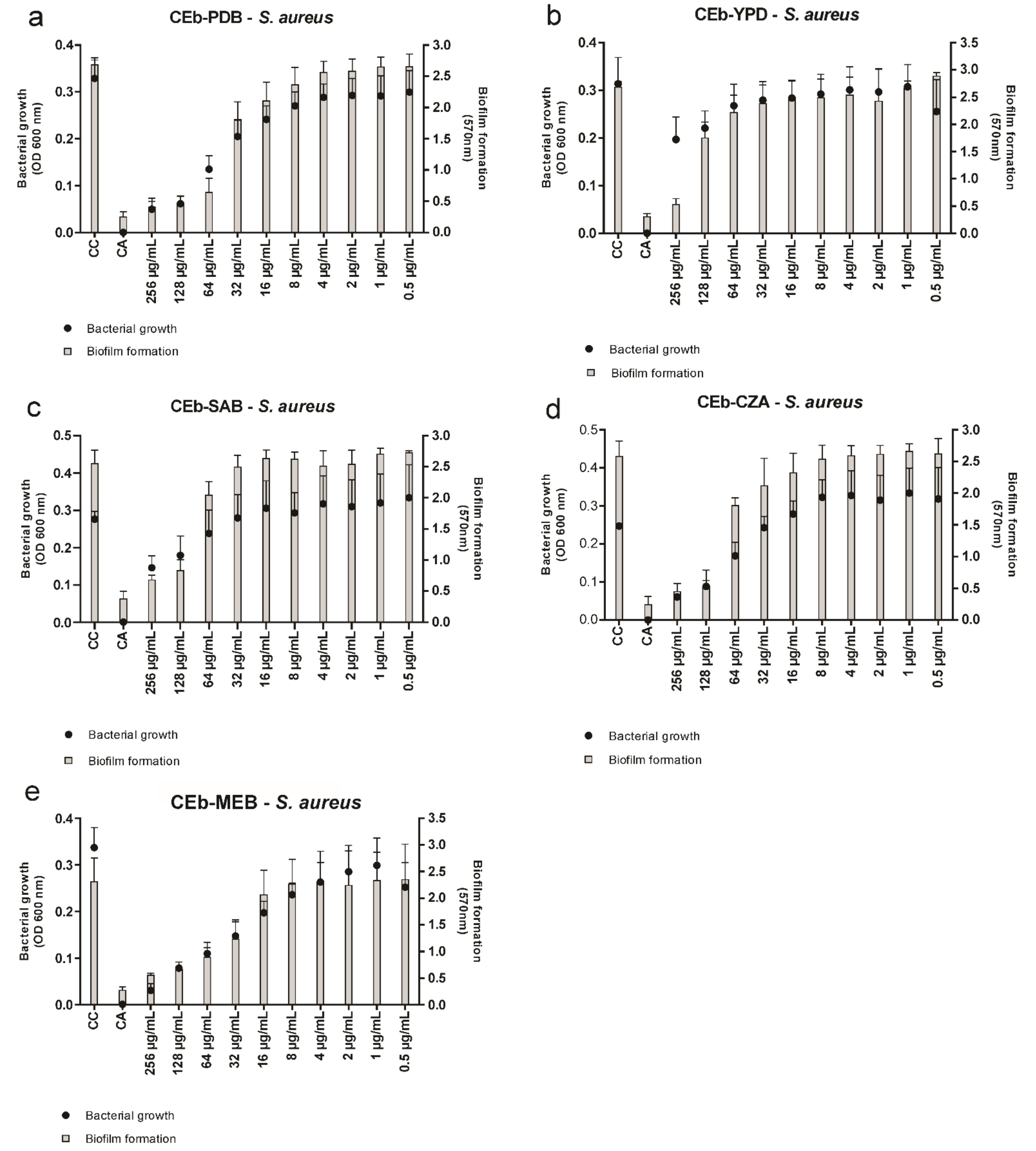

Figure S11. Biofilm formation and bacterial growth of *S. aureus* treated with broth crude extracts of *Penicillium* sp. strain 5MP2F4. (a) CEb-PDB; (b) CEb-YPD; (c) CEb-SAB; (d) CEb-CZA; (e) CEb-MEB) at different concentrations. CC = Growth control, culture medium only; CA = activity control, gentamicin 20 µg/mL.

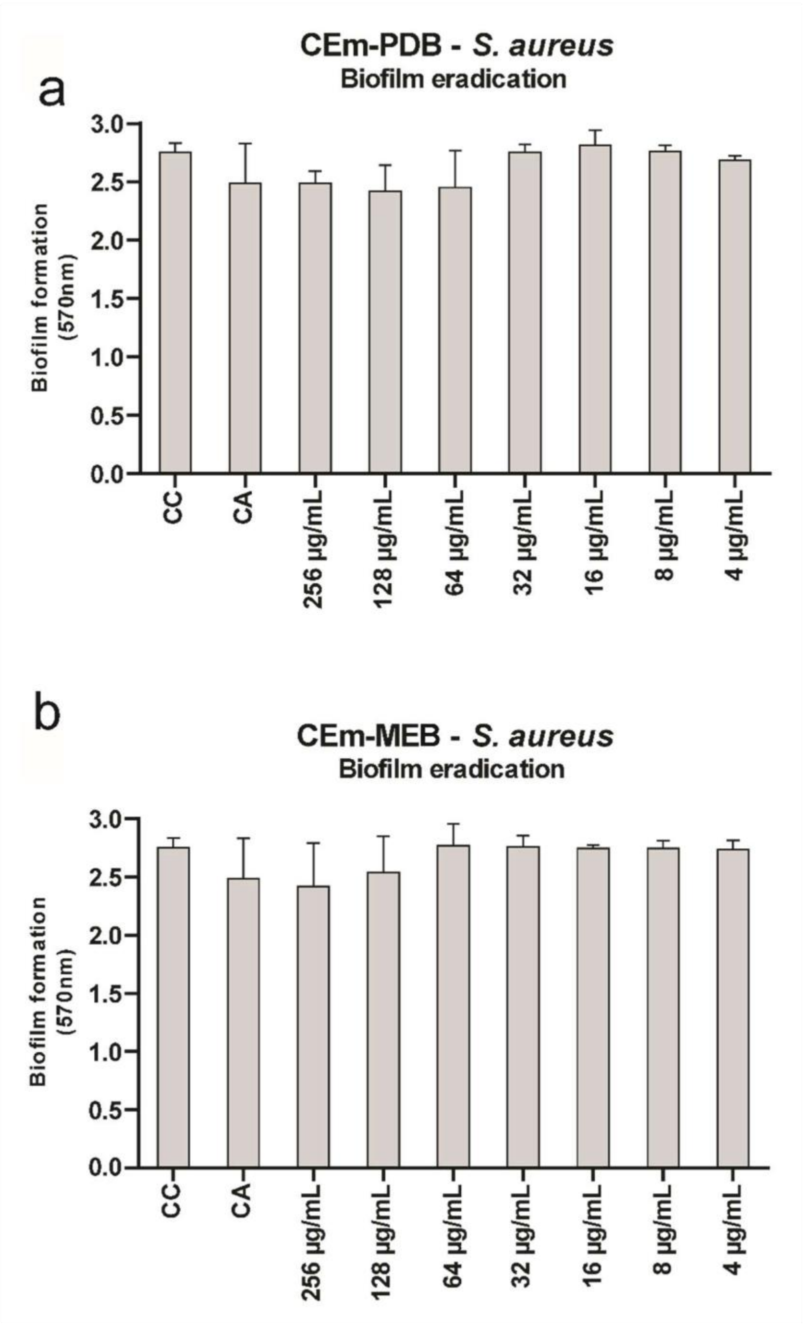

Figure S12. Pre-formed biofilm *S. aureus* treated with the biomass extracts CEm-PDB and CEm-MEB of *Penicillium* sp. strain 5MP2F4 at different concentrations. CC = Growth control; CA = activity control, vancomycin at 20 µg/mL.

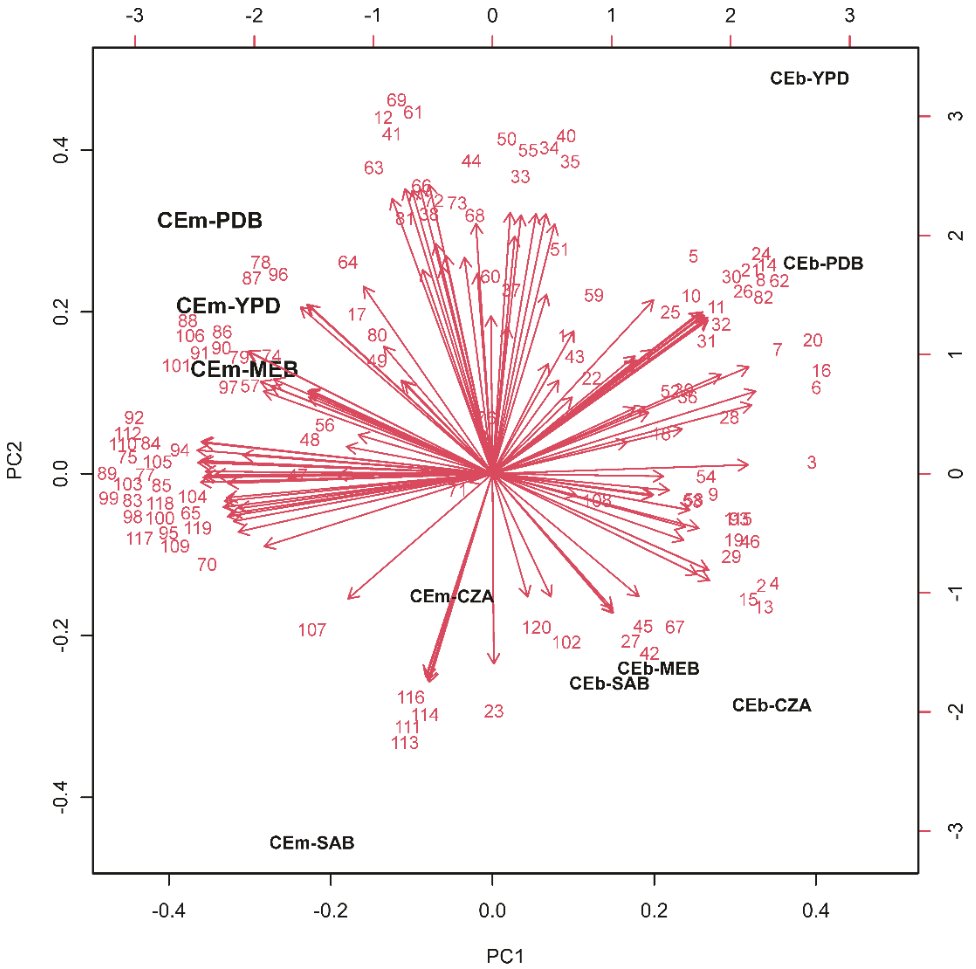

Figure S13. Biplot of principal component 1 (PC1) and principal component 2 (PC2). The samples CEm-PDB, CEm-YPD, and CE-MEB are the most active against *S. aureus*.

.

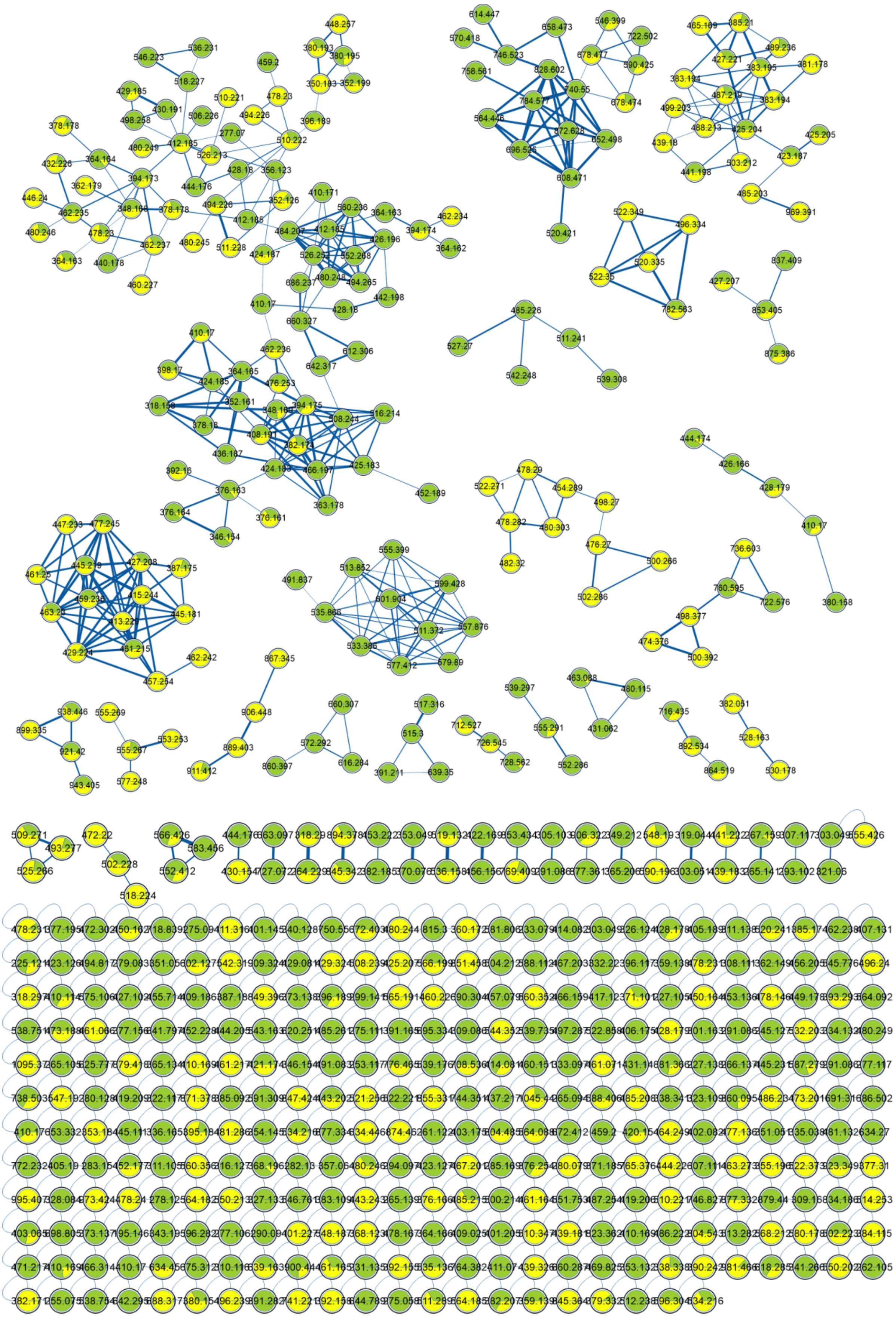

Figure S14. Molecular networking of crude extracts from *Penicillium* sp. strain 5MP2F4, using positive ionization mode (ESI+) data. Crude extracts from broth (CEb) samples are colored green and Crude extracts from biomass (CEm) samples are colored yellow. The edge strength is proportional to the cosine values.

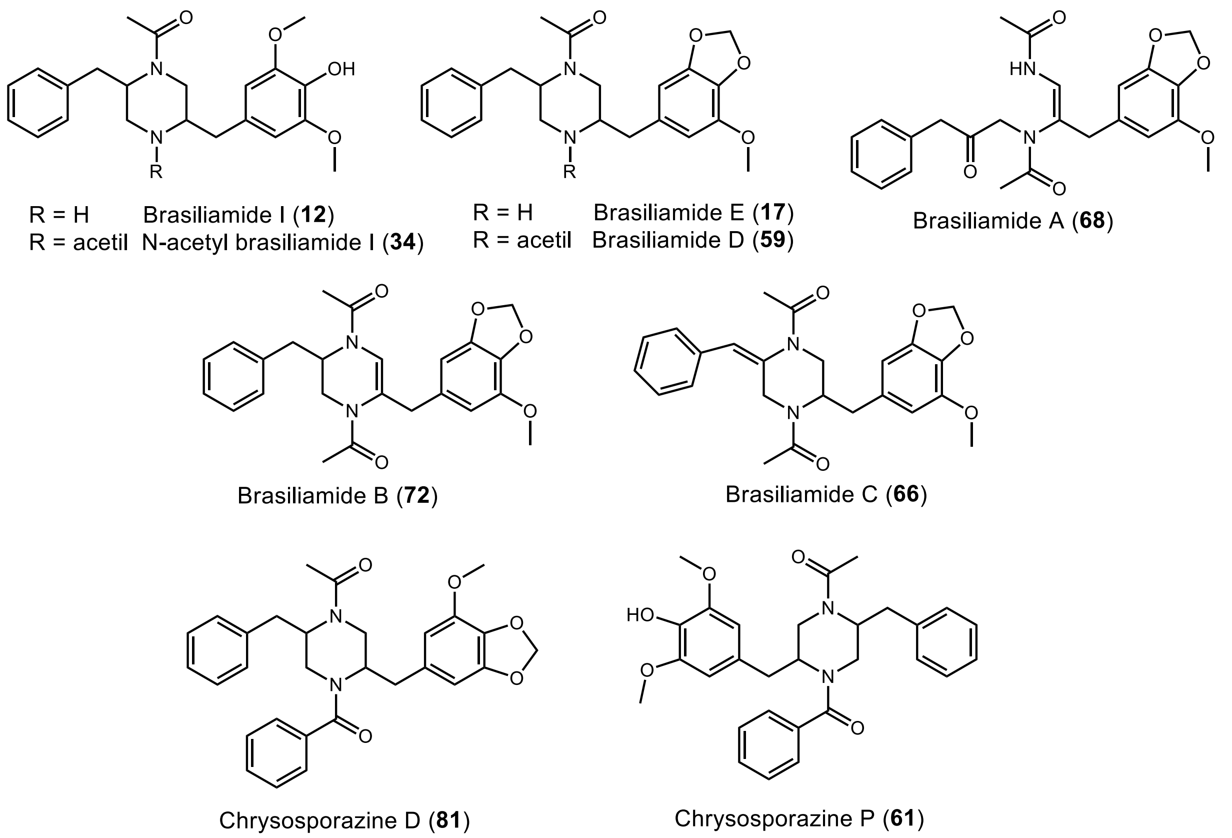

Figure S15. Structures of brasiliamides and chrysosporazines annotated in extracts of *Penicillium* sp. strain 5MP2F4.

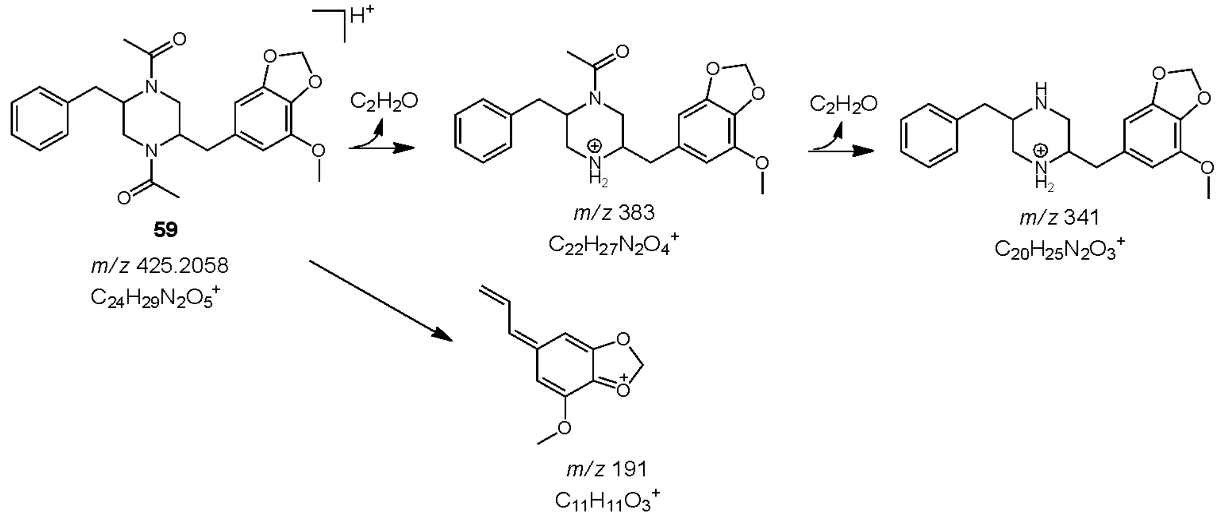

Figure S16. Fragmentation proposal for some observed ions for metabolite **59**.

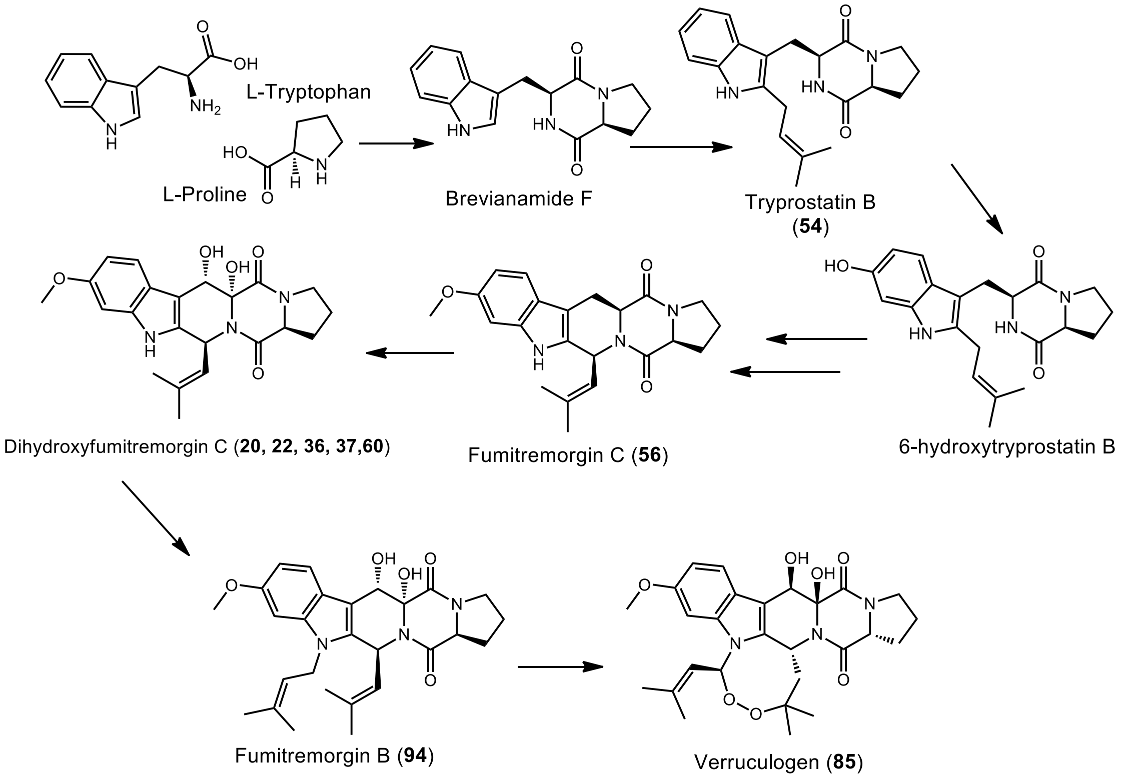

Figure S17. Simplified proposal of the biosynthetic pathway for verruculogen based on Han et all [1]. Metabolites with the indication of numbers were annotated from extracts of *Penicillium* sp. strain 5MP2F4.

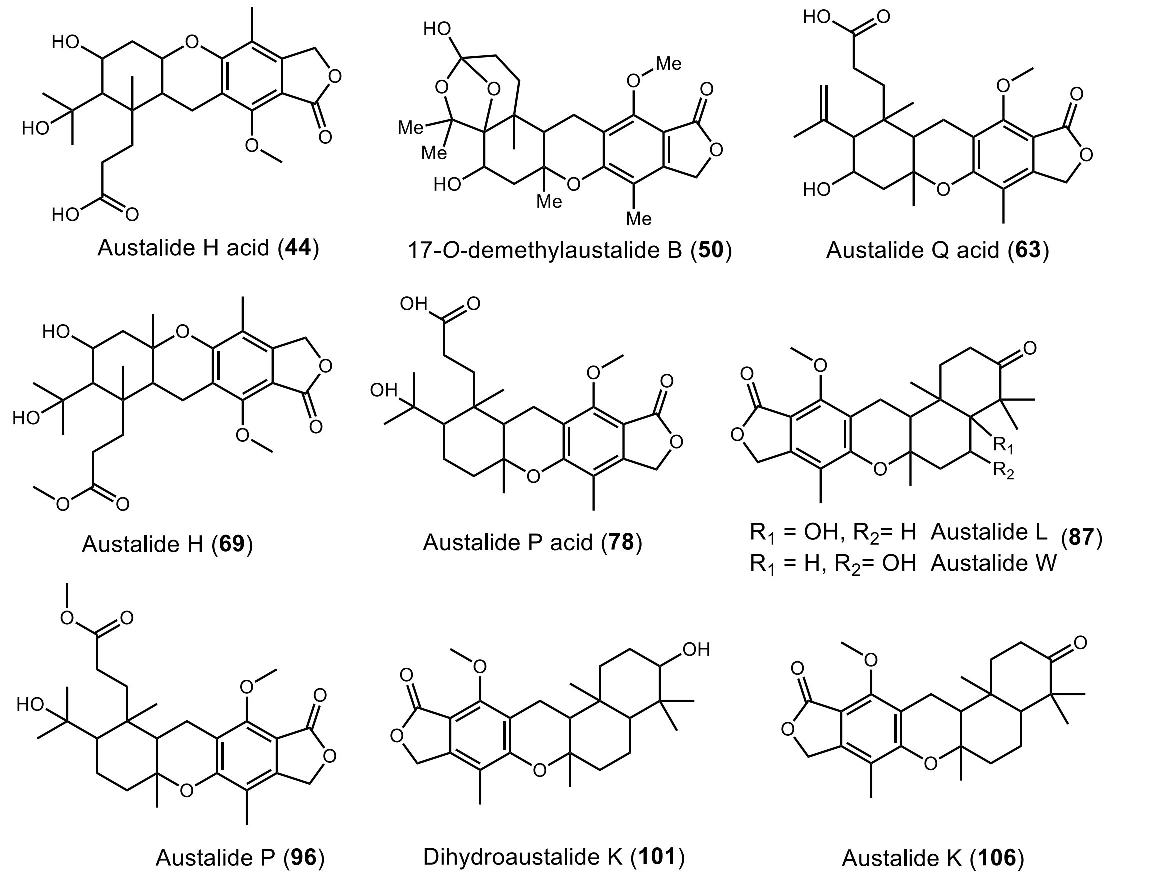

Figure S18. Structures of austalide-type meroterpenoid annotated from extracts of *Penicillium* sp. strain 5MP2F4.

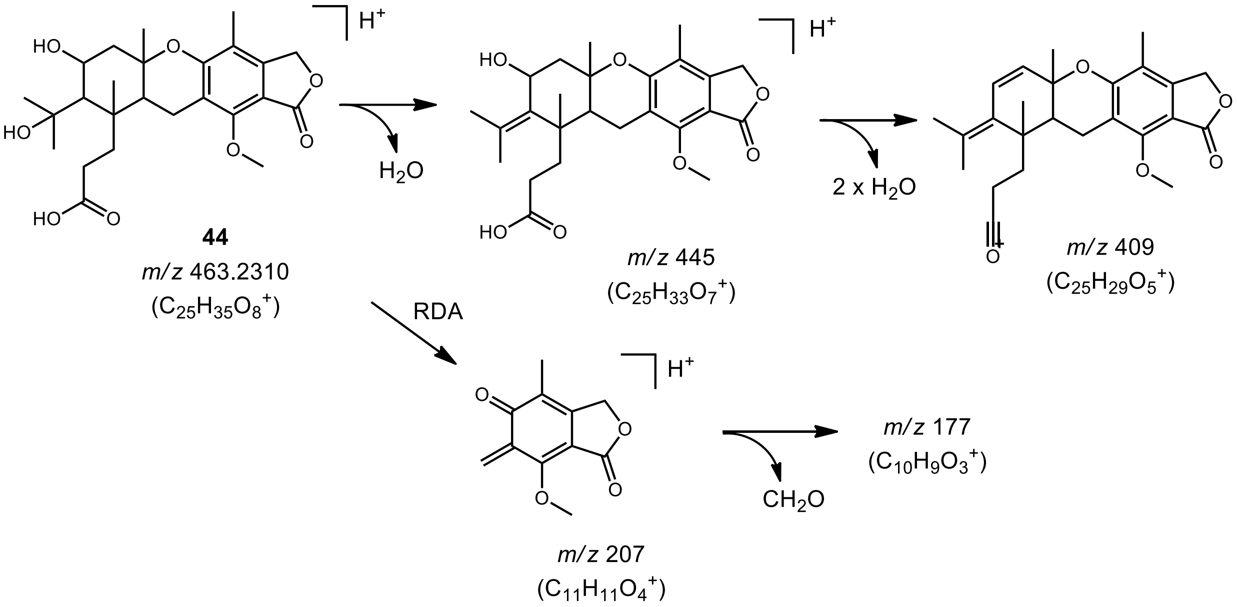

Figure S19. Proposed fragmentation pathway for metabolite **44**.

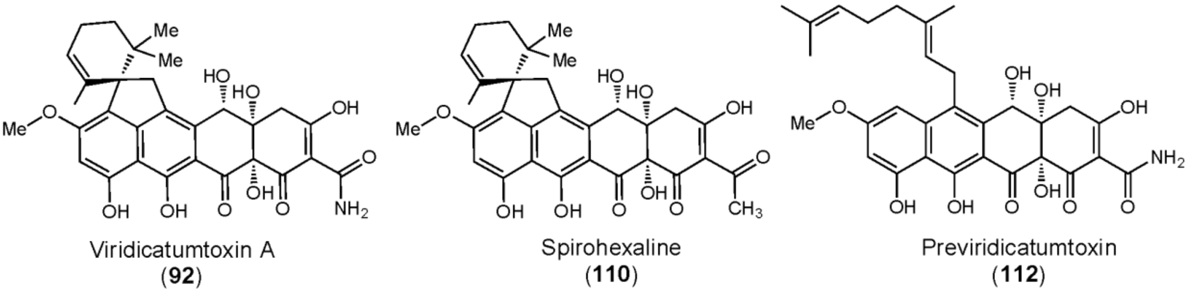

Figure S20. Polyketides annotated from extracts of *Penicillium* sp. strain 5MP2F4.

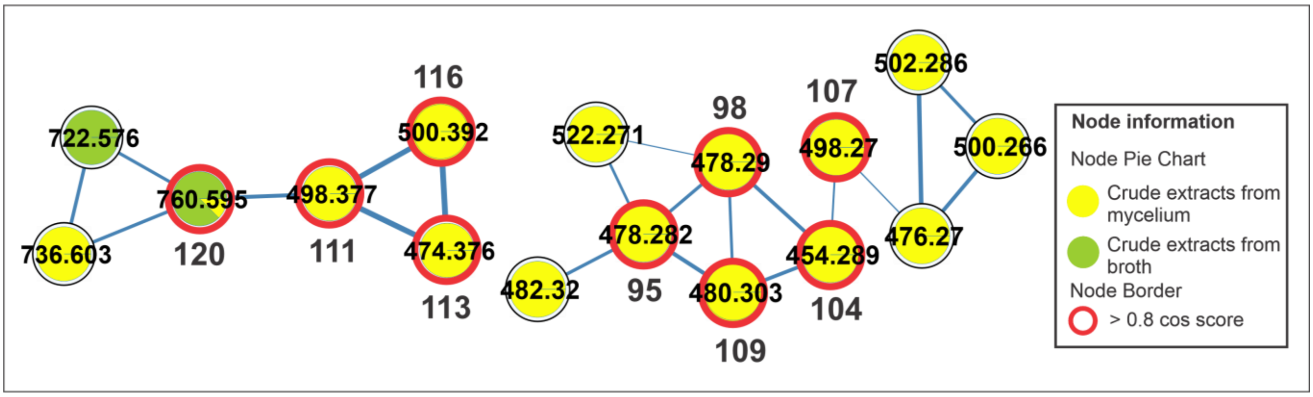

Figure S21. Cluster of lipid compounds from the molecular network of the crude extracts of mycelium biomass (CEm) and broth (CEb) of the endophytic fungi *Penicillium* sp. strain 5MP2F4, considering the positive ionization mode (ESI+) data. Nodes represent parent ions, and edge strength indicates the chemical similarity between the MS/MS spectra. Groups of samples CEm and CEsob in yellow and green color, respectively. The numbers represent the annotated metabolites described in Table 1 and the nodes circled in red indicate the compounds annotated by GNPS.

metabolites MDPI

Table S1: Table of spectral data of constituents of biomass and broth crude extracts from *Penicillium* sp. strain 5MP2F4 by HPLC-DAD-MS/MS.

Peak	RT (min)	UV (nm)	MF**	Positivo (*m/z*)		Negativo (*m/z*)	
				$[M+H]^+$	MS/MS	$[M-H]^-$	MS/MS
1	2.60	301	$C_{14}H_{15}NO_6$	294.0975	210, 164	-	-
2	3.89	-	$C_8H_{12}O_5$	189.0761	171	187.0619	169
3	11.16	-	$C_{11}H_{16}O_7S$	275.0582[#]	209, 171, 158, 153	291.0551	-
4	11.68	-	$C_8H_{10}O_4$	171.0655	155	169.0511	94
5	11.99	-	$C_{11}H_{16}O_7$	261.0985	-	259.0825	-
6	12.90	259, 300	$C_8H_8O_4$	151.0384[#]	-	167.0344	-
7	13.78	298	$C_{15}H_8O_6$	285.0399	-	283.0247	-
8	15.36	-	$C_{11}H_{14}O_6$	243.0866	167	-	-
9	15.65	284	$C_{11}H_{16}N_2O_3$	225.1232	189, 161, 145	223.1092	208
10	16.32	286	$C_{12}H_{18}O_7$	275.1124	257, 207, 197, 165, 153	-	-
11	16.64	280, 320	$C_{10}H_{11}NO_3$	194.0813	176, 158	-	-
12	17.15	290	$C_{22}H_{28}N_2O_4$	385.2123	368, 343, 217, 193, 175, 167	-	-
13	17.26	-	$C_{11}H_{18}N_2O$	195.1486	177, 162, 147	-	-
14	18.54	291	$C_{24}H_{24}N_3O_6$	394.1753[2#]	394, 338, 310, 287, 259, 214, 202, 174	428.1821	-
15	19.34	267, 345	$C_{15}H_{12}O_{10}$	353.0498	322, 261, 246, 221, 205, 177	351.0371	319, 245, 217, 188, 175, 159
16	21.56	-	$C_{12}H_{16}N_2O_4$	207.1115[F]	229, 207, 189, 161, 146	251.1038	-
17	21.73	282	$C_{22}H_{26}N_2O_4$	383.1958	366, 341, 217, 175, 165	-	-
18	22.18	290	$C_{22}H_{23}N_3O_4$	394.1753	-	-	-
19	23.47	-	$C_{19}H_{28}N_2O_4$	349.2129	293, 265, 209, 179, 163	-	-
20	23.78	269, 284	$C_{22}H_{25}N_3O_5$	412.1863	394, 315, 287, 269, 251, 236, 211	-	-
21	24.64	290, 380	$C_{22}H_{25}N_3O_7$	444.1760	390, 362, 339, 311, 230, 202	442.1613	-

22	25.67	268, 296	$C_{22}H_{25}N_3O_5$	412.1855	394, 356, 338, 310, 287, 259, 231, 213, 188	410.1718	-
23	26.21	-	$C_{39}H_{60}N_6O_{15}$	853.4161	741, 569, 457, 379, 285, 173	851.4021	-
24	26.23	290	$C_{24}H_{25}N_7O_3$	410.1701	392, 326, 295, 269, 251, 228,178	458.1934	-
25	26.82	291, 395	$C_{22}H_{25}N_3O_7$	426.1664[#]	391, 362, 319, 293, 267, 230, 202, 174	442.1606	-
26	26.98	290	$C_{22}H_{25}N_3O_6$	410.1705[#]	313, 287, 230, 188, 162	426.1702	-
27	27.06	285, 320	$C_{15}H_{14}O_6$	291.0862	276, 259, 231, 215, 207, 185, 159, 147	289.0718	-
28	27.11	-	$C_{23}H_{29}N_3O_6$	394.1760	297, 279, 269, 251, 236, 224	442.198	-
29	27.14	-	$C_{22}H_{28}O_7$	405.1895	341, 295, 247, 229, 201, 183, 153	403.1743	-
30	27.22	-	$C_{23}H_{29}N_3O_6$	412.1869[M]	370, 315, 287, 258, 216, 193,161	442.1984	-
31	27.36	-	$C_{25}H_{29}N_3O_7$	484.2073	412, 394, 315, 287, 269, 251, 236, 211, 168	-	-
32	28.58	-	$C_{22}H_{23}N_3O_6$	426.1963	343, 287, 269, 211, 167	-	-
33	28.65	290, 390	$C_{22}H_{23}N_3O_5$	410.1705	392, 364, 313, 295, 285, 267, 257, 240, 228, 213, 200, 188	-	-
34	28.76	-	$C_{24}H_{30}N_2O_5$	427.2212	385, 343, 277, 252, 217, 193, 175, 167	-	-
35	28.95	284	$C_{11}H_{16}N_2O_2$	209.1278	-	-	-
36	29.10	284	$C_{22}H_{23}N_3O_4$	394.1745	366, 338, 297, 269, 241	392.1612	-
37	29.38	284	$C_{22}H_{25}N_3O_5$	394.1752[#]	366, 338, 297, 269, 241	410.1714	-
38	29.76	-	$C_{21}H_{23}N_3O_4$	382.1755	358, 251, 236, 221	380.1619	-
39	30.07	-	$C_{22}H_{23}N_3O_4$	394.1756	350, 310, 269, 259, 231, 213, 199	-	-
40	30.13	270	$C_{21}H_{21}N_3O_3$	364.1655	290, 267, 239, 222, 182, 167	-	-
41	30.16	-	$C_{30}H_{34}N_6O_4$	555.2699	397, 354, 217,181	553.2786	-
42	30.42	282	$C_{22}H_{28}O_7$	405.1899	387, 359, 323, 271, 253, 229, 183, 165	403.1753	-
43	30.61	293, 431	$C_{22}H_{23}N_3O_5$	392.1600[#]	337, 313, 267, 251, 197, 179	-	-
44	30.65	269	$C_{25}H_{34}O_8$	463.2310	445, 427, 409, 331, 285, 245, 207, 177	461.2171	-
45	30.65	-	$C_{15}H_{20}O_4$	265.1439	219, 203, 173, 268, 159	263.1289	-
46	30.83	279	$C_{15}H_{22}O_4$	267.1725	249, 203, 185, 175, 159, 147	265.1446	219, 189, 161
47	30.97	290	$C_{22}H_{25}N_3O_4$	378.1790[#]	197, 215, 226, 253, 264, 281, 310, 348	394.1763	-
48	31.01	-	$C_{21}H_{23}N_3O_3$	348.1704[#]	320, 251, 223, 196, 185, 167, 157	364.1665	-
49	31.18	272, 295	$C_{22}H_{23}N_3O_4$	394.1744	350, 297, 259, 231, 213, 198, 174	392.1598	-

50	31.23	270	$C_{25}H_{32}O_8$	461.2156	417, 399, 315, 245, 207, 177	459.2007	-
51	31.28	-	$C_{24}H_{28}N_2O_6$	441.1986	-	-	-
52	31.32	-	$C_{23}H_{25}N_3O_4$	408.1893	390, 251, 236	-	-
53	31.46	267, 345	$C_{22}H_{14}N_4O_8$	463.0870	385, 353, 325, 273, 246, 207, 162	461.0724	-
54	31.49	280	$C_{21}H_{25}N_3O_2$	352.2019	312, 296, 273, 252, 198, 183, 168, 156	-	-
55	31.74	285	$C_{31}H_{41}N_5O_7$	596.3086	568, 457, 356, 317, 280, 231, 259, 181, 169	-	-
56	31.89	274, 298	$C_{22}H_{25}N_3O_3$	380.1947	324, 255, 226, 212, 199, 184	-	-
57	32.08	283	$C_{28}H_{29}N_3O_5$	488.2171	446, 428, 339, 280, 239, 229, 208, 191, 165	-	-
58	32.13	267, 345	$C_{21}H_{18}O_{12}$	431.0600[M]	415, 413, 399	461.0721	
59	32.14	-	$C_{24}H_{28}N_2O_5$	425.2058	383, 341, 250, 217,191, 175	-	-
60	32.37	275	$C_{22}H_{25}N_3O_5$	412.1854	394, 356, 336, 310, 282, 259, 213, 173	-	-
61	32.42	-	$C_{29}H_{32}N_2O_5$	489.2372	471, 447, 429, 311, 263, 238, 207, 193, 167	-	-
62	32.48	-	$C_{21}H_{21}N_3O_2$	348.1698	-	-	-
63	32.48	277	$C_{25}H_{32}O_7$	445.2216	427, 409, 331, 273, 245, 207, 177	-	-
64	32.68	276	$C_{21}H_{25}N_3O_2$	352.2014	296, 284, 268, 240, 198, 170	-	-
65	32.81	-	$C_{27}H_{31}N_3O_7$	510.2220	368, 340, 322, 310, 294, 271, 243, 215, 197, 160	-	-
66	32.89	-	$C_{24}H_{26}N_2O_5$	423.1899	381, 339, 248, 173	-	-
67	32.97	282	$C_{11}H_{18}N_2$	179.1539	-	-	-
68	33.07	275	$C_{24}H_{26}N_2O_6$	439.1861	380, 338, 308, 280, 247, 165	437.1705	378, 214, 172
69	33.19	278	$C_{26}H_{36}O_8$	477.2460	459, 441, 409, 391, 345, 318, 245, 207, 177	-	-
70	33.29	-	$C_{33}H_{36}N_2O_3$	509.2747	450, 340, 322, 294, 283, 271, 243, 215, 160	-	-
71	33.33	-	$C_{27}H_{31}N_3O_7$	510.2316	340, 322, 294, 271, 243, 215, 197, 160	-	-
72	33.50	272	$C_{24}H_{26}N_2O_5$	423.1865	363, 189, 165	-	-
73	33.58	269	$C_{25}H_{32}O_7$	445.2198	369, 331, 273, 245 ,207, 177	-	-
74	33.90	-	$C_{29}H_{30}N_2O_6$	503.2177	446, 382, 341, 279, 238, 191, 175, 165	-	-
75	34.06	-	$C_{27}H_{31}N_3O_7$	510.2231	322, 279, 271, 243, 225, 201	-	-
76	34.45	-	$C_{21}H_{23}N_3O_5$	398.1712	313, 295, 267, 252, 239, 224, 209	396.1581	-
77	34.56	-	$C_{18}H_{32}O_4$	335.2181[N]	219, 199, 183, 171	311.2223	-
78	34.60	269	$C_{25}H_{34}O_7$	447.2362	329, 273, 247, 207, 177	445.2219	-

79	34.64	273	$C_{29}H_{30}N_2O_6$	503.2249	444, 382, 340, 238, 207, 177, 165, 149	-	-
80	34.67	-	$C_{22}H_{23}N_3O_5$	410.1712	313, 295, 267, 239, 224	-	-
81	34.73	-	$C_{29}H_{30}N_2O_5$	487.2217	469, 445, 366, 335, 279, 238, 191, 165	-	-
82	34.84	-	$C_{37}H_{46}N_3O_8$	660.3290	412, 394, 366, 315, 287	-	-
83	35.09	-	$C_{27}H_{33}N_3O_8$	510.2213#	381, 320, 292, 252, 227, 157	526.217	-
84	35.09	-	$C_{22}H_{31}N_5O_7$	478.2324	376, 336, 322, 269, 243, 215, 211, 197	-	-
85	35.38	276, 295	$C_{27}H_{33}N_3O_7$	494.2282#	352, 255, 227, 199	510.2234	-
86	35.59	284	$C_{27}H_{33}N_3O_5$	462.2375#	411, 356, 327, 282, 231, 214, 148	478.2321	-
87	35.81	265	$C_{25}H_{32}O_6$	429.2259	354, 329, 273, 245, 207, 177, 149	-	-
88	35.93	286	$C_{29}H_{28}N_2O_5$	485.2076	380, 322, 218, 165	-	-
89	36.21	289	$C_{27}H_{33}N_3O_4$	446.2426#	362, 226, 212, 160	-	-
90	36.34	284	$C_{27}H_{33}N_3O_6$	478.2325#	432, 364, 279, 240, 224, 212, 198	-	-
91	36.41	277, 296	$C_{27}H_{33}N_3O_5$	462.2375#	320, 268, 240, 226, 212, 186	-	-
92	36.55	284, 430	$C_{30}H_{31}NO_{10}$	548.1909#	531, 513, 464, 433, 421, 407, 393, 375, 365, 335, 279	564.187	-
93	36.55	-	$C_{37}H_{43}N_3O_7$	642.3179	394, 348, 269, 297, 251	-	-
94	36.58	290	$C_{27}H_{33}N_3O_5$	462.2389#	394, 327, 310, 269, 240, 214, 200, 174, 158	-	-
95	36.68	284	$C_{23}H_{44}NO_7P$	478.2869	337, 306, 191	476.2777	-
96	36.77	-	$C_{26}H_{36}O_7$	443.2418#	329, 273, 245, 207, 177, 149	-	-
97	36.83	-	$C_{30}H_{31}NO_{10}$	548.1909#	531, 513, 439, 421, 393, 363, 335, 325, 305, 283, 278	-	-
98	37.02	287	$C_{23}H_{44}NO_7P$	478.2900	337, 306	476.2744	-
99	37.05	-	$C_{27}H_{33}N_3O_3$	448.2574	396, 380, 281, 255, 226, 199	-	-
100	37.25	-	$C_{26}H_{50}NO_7P$	520.3381	337, 244, 152	-	-
101	37.32	-	$C_{25}H_{34}O_5$	415.2484	359, 261, 207, 177	-	-
102	37.42	-	$C_{28}H_{57}NO_9$	552.4114	439, 394, 326, 281, 227, 213, 199, 185	-	-
103	37.57	-	$C_{27}H_{31}N_3O_4$	462.2378	-	-	-
104	37.74	-	$C_{21}H_{44}NO_7P$	454.2941	313, 282	452.2788	-
105	37.93	-	$C_{27}H_{31}N_3O_3$	446.2434	378, 363, 350, 311, 294, 281, 253, 243, 212	-	-
106	38.04	-	$C_{25}H_{32}O_5$	413.2400	354, 261, 207, 177	-	-

107	38.07	-	$C_{27}H_{52}N_3O_3P$	498.3781	236	-	-
108	38.13	-	$C_{30}H_{55}N_5O_5$	566.4266	340, 295, 267, 227, 199	-	-
109	38.28	-	$C_{23}H_{47}NO_7P$	480.3082	449, 339, 308	478.2944	-
110	38.40	-	$C_{31}H_{32}O_{10}$	547.1949#	477, 435, 365, 313, 267, 221, 184	563.1918	-
111	38.45	-	$C_{28}H_{51}NO_6$	498.3711	480, 236	-	-
112	38.72	-	$C_{30}H_{33}NO_{10}$	550.2062#	515, 431, 409, 351, 313, 275, 257	566.2027	-
113	39.35	-	$C_{26}H_{51}NO_6$	474.3750	313, 236	-	-
114	39.43	-	$C_{28}H_{53}NO_6$	500.3936	236	-	-
115	39.83	-	$C_{30}H_{44}O_7$	517.3159	454, 357, 233, 203, 187	515.3009	-
116	39.85	-	$C_{28}H_{53}NO_6$	500.3881	482, 339, 236	-	-
117	41.05	-	$C_{23}H_{36}O_4$	377.2712	-	-	-
118	41.15	-	$C_{23}H_{42}N_2O_2$	379.3334	-	-	-
119	42.28	-	$C_{18}H_{32}O_2$	303.2285N	234	-	-
120	42.29	-	$C_{46}H_{81}NO_7$	760.6030	653, 600, 498, 480, 436, 236	-	-

MF: Molecular Formula; RT: retention time; *: confirmed by authentic standard; **determined by considering errors and mSigma less than 8 ppm and 30, respectively. #: $[M+H-H_2O]^+$, N: $[M+Na]^+$, F: $[M+H-HCOOH]^+$, M: $[M+H-CH_3OH]^+$.

metabolites

Table S2. Presence or absence of compounds in the extracts of broth and biomass of *Penicillium* sp. strain 5MP2F4 by HPLC-DAD-MS analysis.

Peak	RT	CEb-CZAb	CEb-SAB	CEb-MEB	CEb-PDB	CEb-YPD	CEm-CZA	CEm-SAB	CEm-MEB	CEm-PDB	CEm-YPD
1	2.60	-	-	X	X	X	X	X	X	X	-
2	3.89	X	X	X	X	X	-	X	X	-	-
3	11.17	X	X	-	X	X	-	-	-	-	-
4	11.68	X	X	X	X	X	X	X	X	X	-
5	12.00	X	-	X	X	X	-	-	-	X	X
6	12.90	X	-	-	X	X	-	-	-	-	-
7	13.79	-	-	X	X	X	-	-	-	-	-
8	15.36	-	-	-	X	X	-	-	-	-	-
9	15.65	X	X	X	X	X	-	X	X	X	-
10	16.33	X	X	X	X	X	-	X	X	X	X
11	16.64	-	-	-	-	X	-	-	-	-	-
12	17.16	-	-	-	X	X	-	-	X	X	X
13	17.26	X	X	X	X	X	-	X	X	-	-
14	18.54	-	-	-	X	X	-	-	-	-	-
15	19.35	X	-	X	X	-	-	-	-	-	-
16	21.56	X	-	-	X	X	-	-	-	-	-
17	21.74	-	-	-	X	-	-	-	X	X	-
18	22.18	-	X	-	-	X	-	-	-	-	-
19	23.48	X	-	-	X	-	-	-	-	-	-
20	23.78	X	-	-	X	X	-	-	-	-	-
21	24.64	-	-	-	X	X	-	-	-	-	-
22	25.67	-	X	X	X	X	-	X	-	X	X
23	26.21	-	X	-	-	-	-	X	-	-	-
24	26.24	-	-	-	X	X	-	-	-	-	-
25	26.82	-	-	-	-	X	-	-	-	-	-
26	26.99	-	-	-	X	X	-	-	-	-	-
27	27.07	X	-	-	-	-	-	-	-	-	-
28	27.11	X	-	-	-	X	-	-	-	-	-
29	27.15	X	-	-	X	-	-	-	-	-	-
30	27.22	-	-	-	X	X	-	-	-	-	-
31	27.36	-	-	-	-	X	-	-	-	-	-
32	28.58	-	-	-	-	X	-	-	-	-	-
33	28.66	-	X	-	X	X	-	-	X	X	X
34	28.76	-	-	-	X	X	-	-	-	X	X
35	28.95	-	-	-	X	X	-	-	X	X	-

36	29.10	X	-	-	X	X	-	X	-	X	-
37	29.38	X	X	-	X	X	-	X	X	X	X
38	29.76	-	-	-	-	X	-	-	-	X	X
39	30.08	-	-	-	X	X	-	X	-	-	-
40	30.14	-	-	-	X	X	-	-	-	X	X
41	30.16	-	-	-	X	X	-	-	X	X	X
42	30.43	X	-	-	-	-	-	-	-	-	-
43	30.62	-	-	-	X	X	-	X	-	-	X
44	30.66	X	-	-	X	X	-	-	X	X	X
45	30.66	X	-	-	-	-	-	-	-	-	-
46	30.83	X	X	X	X	X	X	X	X	X	-
47	30.97	-	X	X	-	X	-	X	X	X	X
48	31.01	-	X	X	-	X	-	X	X	X	X
49	31.19	-	X	X	X	X	-	X	X	X	X
50	31.23	X	X	X	X	X	X	X	X	X	X
51	31.29	X	-	-	X	X	-	-	X	X	-
52	31.32	X	-	-	X	X	-	X	-	X	-
53	31.46	-	-	X	X	-	-	-	-	-	-
54	31.49	X	X	X	X	X	-	X	X	X	-
55	31.74	X	X	X	X	X	X	X	X	X	X
56	31.89	-	X	X	X	X	-	X	X	X	X
57	32.08	-	-	-	-	-	-	-	X	X	-
58	32.13	-	-	X	X	-	-	-	-	-	-
59	32.14	X	-	X	X	X	-	-	X	X	-
60	32.37	-	-	-	X	X	-	X	-	X	X
61	32.43	-	-	-	X	X	-	-	X	X	X
62	32.48	-	-	-	X	X	-	-	-	-	-
63	32.48	-	-	-	X	X	-	-	X	X	X
64	32.69	-	-	-	X	X	-	X	X	X	X
65	32.82	-	X	-	-	-	-	X	X	X	X
66	32.90	X	-	-	X	X	-	-	X	X	X
67	32.97	X	X	X	X	X	-	X	X	X	-
68	33.08	X	-	X	X	X	X	-	X	X	X
69	33.19	-	-	-	X	X	-	-	X	X	X
70	33.29	X	-	-	-	-	X	X	X	X	X
71	33.34	-	X	X	X	X	-	X	X	-	X
72	33.50	-	-	-	X	X	-	-	X	X	-
73	33.58	X	-	X	X	X	-	-	X	X	X
74	33.91	-	-	-	-	-	-	-	X	X	-
75	34.07	-	-	-	-	-	-	X	X	X	X
76	34.45	-	-	-	-	X	-	X	-	-	X

77	34.56	-	-	-	-	-	X	X	X	X	X
78	34.61	-	-	-	X	-	-	-	X	X	X
79	34.65	-	-	-	-	-	-	-	X	X	-
80	34.68	-	-	-	-	X	-	X	-	X	X
81	34.74	X	-	-	X	X	X	-	X	X	X
82	34.84	-	-	-	X	X	-	-	-	-	-
83	35.09	-	-	-	-	-	-	X	X	X	X
84	35.09	X	X	X	X	X	X	X	X	X	X
85	35.39	X	X	X	X	X	X	X	X	X	X
86	35.59	-	-	-	-	X	-	X	X	X	X
87	35.82	-	-	-	X	-	-	-	X	X	X
88	35.94	-	-	-	-	-	-	-	X	X	X
89	36.21	-	-	-	-	-	-	X	X	X	X
90	36.35	-	-	-	-	X	-	X	X	X	X
91	36.41	-	-	-	-	X	-	X	X	X	X
92	36.55	X	X	X	X	X	X	X	X	X	X
93	36.55	X	-	-	X	-	-	-	-	-	-
94	36.58	X	X	X	X	X	X	X	X	X	X
95	36.68	-	-	-	-	-	X	X	X	X	X
96	36.78	-	-	-	X	-	-	-	X	X	X
97	36.83	-	-	-	-	-	-	-	X	X	-
98	37.02	-	-	-	-	-	X	X	X	X	X
99	37.05	-	-	-	-	-	-	X	X	X	X
100	37.26	-	-	-	-	-	X	X	X	X	X
101	37.33	-	-	-	-	-	X	-	X	X	X
102	37.43	-	X	X	X	-	-	X	X	-	-
103	37.57	X	X	X	X	X	X	X	X	X	X
104	37.75	-	-	-	-	-	X	X	X	X	X
105	37.93	-	-	-	-	-	-	X	X	X	X
106	38.04	-	-	-	-	-	-	-	X	X	X
107	38.07	-	-	-	-	-	-	X	-	X	-
108	38.13	X	X	X	X	X	-	X	X	X	X
109	38.29	-	-	-	-	-	X	X	X	X	X
110	38.40	-	-	-	-	-	-	X	X	X	X
111	38.46	-	-	-	-	-	-	X	-	-	-
112	38.73	-	-	-	-	-	-	X	X	X	X
113	39.36	-	-	-	-	-	-	X	-	-	-
114	39.44	-	-	-	-	-	-	X	-	-	-
115	39.83	X	-	-	X	-	-	-	-	-	-
116	39.86	-	-	-	-	-	-	X	-	-	-
117	41.06	-	-	-	-	-	X	X	X	X	X

118	41.15	-	-	-	-	-	X	X	X	X	X
119	42.29	-	-	-	-	-	X	X	X	X	X
120	42.30	X	X	-	-	-	-	-	X	-	-

References

1. Han, J.; Liu, M.; Jenkins, I.D.; Liu, X.; Zhang, L.; Quinn, R.J.; Feng, Y. Genome-inspired chemical exploration of marine fungus *Aspergillus fumigatus* MF071. *Mar. Drugs* **2020**, *18*, 352.
